# A Critical Role for the Mitochondrial Pyruvate Carrier in Hepatic Stellate Cell Activation

**DOI:** 10.1016/j.jcmgh.2025.101517

**Published:** 2025-04-14

**Authors:** Mohammad Habibi, Daniel Ferguson, Sophie J. Eichler, Mandy M. Chan, Christina Fu, Terri A. Pietka, Andrea L. Bredemeyer, Andrew LaPoint, Trevor M. Shew, Mai He, Kim H.H. Liss, Andrew J. Lutkewitte, Kevin Cho, Joel D. Schilling, Gary J. Patti, Brian N. Finck

**Affiliations:** 1 Department of Medicine, Washington University School of Medicine, St. Louis, Missouri; 2 Department of Pathology and Immunology, Washington University School of Medicine, St. Louis, Missouri; 3 Department of Pediatrics, Washington University School of Medicine, St. Louis, Missouri; 4 Department of Internal Medicine, University of Kansas Medical Center, Kansas City, Kansas; 5 Department of Chemistry, Washington University, St. Louis, Missouri

**Keywords:** Collagen, Fibrosis, HIF1alpha, MASLD, TCA Cycle

## Abstract

**Background & Aims:**

Hepatic stellate cells (HSCs) are non-parenchymal cells of the liver that produce the extracellular matrix that forms fibrotic lesions in chronic liver disease, including metabolic dysfunction-associated steatohepatitis (MASH). The mitochondrial pyruvate carrier (MPC) catalyzes the transport of pyruvate from the cytosol into the mitochondrial matrix, which is a critical step in pyruvate metabolism. An MPC inhibitor has shown promise as a novel therapeutic for MASH and HSC activation, but a mechanistic understanding of the direct effects of MPC inhibition on HSC activation is lacking.

**Methods:**

Stable lines of LX2 cells expressing short hairpin RNA against MPC2 were established and examined in a series of studies to assess HSC metabolism and activation. Mice with conditional, HSC-specific MPC2 deletion were generated and their phenotypes assessed in the context of diets that cause hepatic steatosis, injury, and early-stage fibrosis.

**Results:**

Genetic suppression of MPC activity markedly decreased expression of markers of HSC activation in vitro. MPC knockdown reduced the abundance of several intermediates of the tricarboxylic acid cycle and attenuated HSC activation by suppressing hypoxia inducible factor-1α signaling. Supplementing alpha-ketoglutarate to replenish the tricarboxylic acid cycle intermediates was sufficient to overcome the effects of MPC inhibition on hypoxia inducible factor-1α and HSC activation. On high-fat diets, mice with HSC-specific MPC deletion exhibited reduced circulating transaminases, numbers of HSCs, and hepatic expression of markers of HSC activation and inflammation compared with wild-type mice.

**Conclusions:**

These data suggest that MPC inhibition modulates HSC metabolism to attenuate activation and illuminate mechanisms by which MPC inhibitors could prove therapeutically beneficial for treating MASH.

## Graphical abstract

**Figure:**
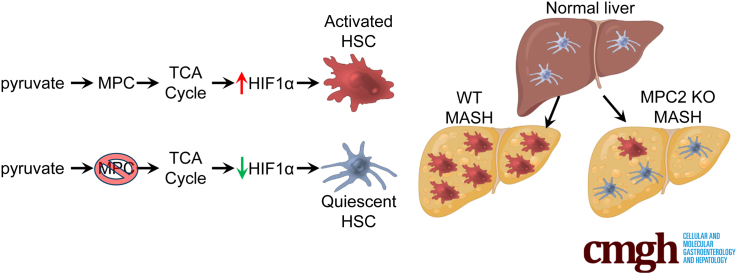

**Summary**

Activated hepatic stellate cells are the primary mediators of the development of fibrosis in a variety of chronic liver diseases. Genetic inhibition of the mitochondrial pyruvate carrier, which catalyzes an important step in intermediary metabolism, suppresses hepatic stellate cell activation. These studies identify new metabolic mechanisms that regulate stellate cell function and highlight the potential of targeting mitochondrial metabolism for treating fibrotic liver disease.

The incidence of metabolic dysfunction-associated steatotic liver disease (MASLD) and steatohepatitis (MASH) is growing rapidly. MASH is characterized by inflammation and fibrosis and dramatically increases the risk of developing cirrhosis, liver failure, and hepatocellular carcinoma.^1^, ^2^, ^3^, ^4^ Despite being a leading cause of liver-related morbidity and mortality, there is only a single, recently-licensed drug therapy for MASH in the United States, and many of the molecular mechanisms driving the progression of MASLD to MASH are unclear.^5^ The liver is composed of a variety of cell types, including parenchymal (hepatocytes) and non-parenchymal cells (cholangiocytes, endothelial, immune, and stellate cells). Although multiple cell lineages contribute to MASH progression, hepatic stellate cells (HSCs) are the primary source of extracellular matrix production and fibrosis in MASH and a variety of chronic liver diseases.^6^

In their inactive (quiescent) state, HSCs display an adipogenic-like transcriptional program and are a major storage depot for vitamin A in the form of retinyl ester-rich lipid droplets.^7^ In response to hepatic injury, HSCs can proliferate, migrate to sites of injury, and differentiate into myofibroblasts that secrete components of the extracellular matrix that make up fibrotic lesions. HSCs are activated in response to a variety of stimuli including factors released in response to cell injury and death, changes in the extracellular matrix, and signals from hepatocytes and immune cells, including numerous cytokines and growth factors.^8^ Although important for tissue repair, chronic activation of HSCs leads to excess extracellular matrix protein (e.g., collagen 1 and collagen 3) deposition in the liver, resulting in hepatic scarring (fibrosis).^9^ Hepatic fibrosis is the most reliable known predictor of progression to more severe forms of MASH, MASH-related liver failure, and all-cause mortality.^10^^,^^11^

Importantly, activated HSCs undergo dramatic changes in metabolism to meet the high energy demand necessary for proliferation and extracellular matrix (ECM) production.^12^ Upon HSC activation, glucose uptake is enhanced and there are marked increases in rates of anaerobic glycolysis.^13^ Activated HSCs are also highly reliant on glutaminolysis, the breakdown of glutamine to α-ketoglutarate (αKG), as a fuel source for the tricarboxylic acid (TCA) cycle.^14^ Prior work has demonstrated that enhanced glucose uptake and glycolysis is required for fibroblasts to sustain transforming growth factor β (TGF-β)-induced collagen synthesis,^15^^,^^16^ and interventions to suppress these metabolic changes have been shown to result in reduced HSC activation.^13^^,^^17^ Based on the importance of energy metabolism in HSC function, it seems plausible that these pathways may be targeted therapeutically to limit the development and progression of fibrosis.

Previous work from our lab has suggested that an inhibitor of the mitochondrial pyruvate carrier (MPC) can suppress markers of HSC activation and prevent or reverse fibrosis in a mouse model of MASH.^18^ The MPC plays a pivotal role in mitochondrial metabolism by transporting pyruvate, a product of glycolysis, into the mitochondrial matrix to enter the TCA cycle (Figure 1*A*). The MPC is a heterodimeric complex composed of the MPC1 and MPC2 proteins.^19^^,^^20^ Both subunits are required for MPC activity and stability, as the deletion of either protein destabilizes the complex.^19^, ^20^, ^21^ MPC inhibition in multiple tissues provides metabolic benefits^21^^,^^22^ and reduces MASH progression in mice.^18^ Furthermore, an MPC inhibitor showed some beneficial effects in a Phase IIb clinical trial for treating MASH.^23^ However, little is known about the direct effects of MPC inhibition on HSC metabolism and activation, and the molecular mechanisms mediating these effects are poorly understood. To investigate this, we examined how disrupting the MPC affects stellate cell metabolism and activation in vitro, and generated mice with HSC-specific *Mpc2* deletion to understand the effects in vivo. Here, we show that genetic inhibition of the MPC attenuates the activation of HSCs due to impaired TCA cycle activity and suppression of hypoxia inducible factor 1α (HIF1α) signaling and collagen gene expression. Furthermore, we demonstrate that MPC deletion in HSCs protects mice from HSC activation and inflammation in the context of diets that cause hepatic steatosis and liver injury.

**Figure 1:**
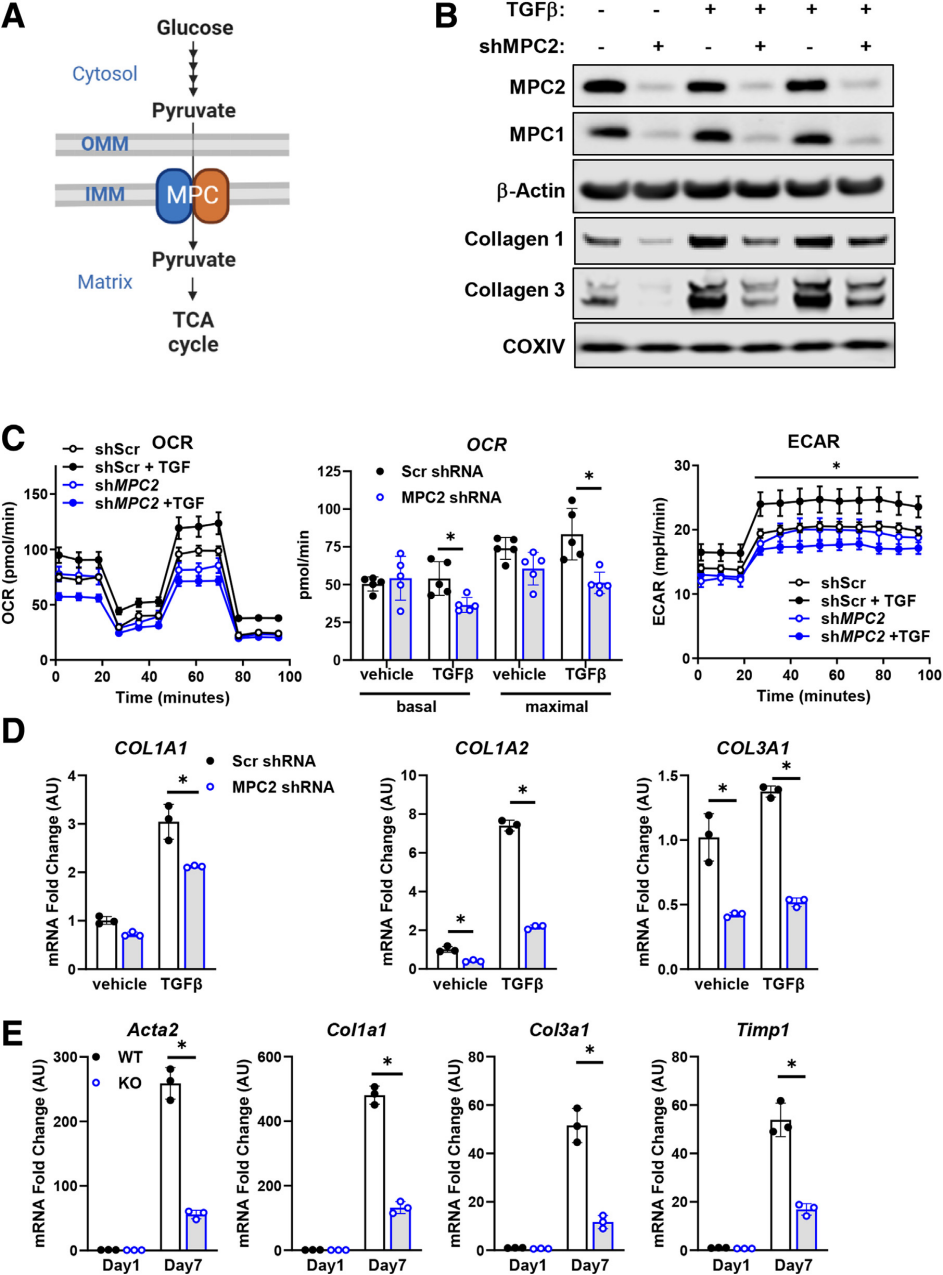
**Genetic inhibition of the MPC reduces hepatic stellate cell activation in vitro.** (*A*) A schematic for glucose metabolism and pyruvate entry into the mitochondrial TCA cycle is shown. IMM, inner mitochondrial membrane; OMM, outer mitochondrial membrane. Created with BioRender.com. (*B*) Stable human LX2 hepatic stellate cells were engineered to express shRNA against MPC2 (or Scr shRNA control). TGF-β-1 (5 ng/mL) was added to culture medium of some wells to activate HSC. Collagen or MPC proteins were detected by Western blotting. (*C*) Seahorse respirometry assays demonstrate that OCR and extracellular acidification rate (ECAR) are decreased by MPC2 shRNA. (*D*) Collagen isoform gene expression was decreased in activated LX2 cells expressing MPC2 shRNA. Data are expressed as mean ± SEM, relative to control cells expressing non-targeting shRNA. ∗*P* < .05 vs Scr shRNA cells of the same treatment group. (*E*) *Col1a1*, *Col3a1*, *Acta2*, and *Timp1* gene expression of isolated HSCs from WT *Mpc2*^*fl/fl*^ mice and Lrat-*Mpc2*^*-/-*^ littermates that were cultured for 7 days (Day 7). Some cells were harvested after 1 day of culture (Day 1) for quiescent HSCs. Gene expression was measured by RT-qPCR, and data are expressed as mean ± SEM, relative to Day 1 HSC. ∗*P* < .01 vs WT day 7 cells as determined by 1-way ANOVA, followed by Tukey’s *post hoc* analysis. n = 3 technical replicates from a representative experiment.

## Results

### Inhibition of the MPC Attenuates HSC Activation In Vitro

To test the role of MPC inhibition on HSC activation, we generated a stable line of LX2 human HSC cells expressing short hairpin RNAs (shRNAs) against MPC2 and compared them to a control line expressing scrambled (Scr) shRNA. LX2 cells were activated by treating them with TGF-β. As expected, cells expressing MPC2 shRNA exhibited a concomitant reduction in MPC2 and MPC1 protein abundance due to the destabilization of the MPC complex (Figure 1*B*). Seahorse analyses demonstrated that rates of oxygen consumption were diminished in cells with MPC2 knockdown compared with shScr controls in a standardized stress test (Figure 1*C*). Interestingly, glycolysis, as measured by extracellular acidification rates, was also diminished by MPC2 knockdown under TGF-β-stimulated conditions. LX2 cells with MPC knockdown exhibited lower expression of collagen mRNA and protein compared with scrambled control cells at baseline and after stimulation with TGF-β (Figure 1*B, D*).

To further investigate the role of the MPC in HSCs, we generated mice with stellate cell-specific deletion of *Mpc2* by crossing *Mpc2*^*fl*/*fl*^ mice with mice expressing Cre recombinase under the lecithin-retinol acyltransferase (Lrat-Cre) promoter^6^ to generate Lrat-*Mpc2*^-/-^ mice. Primary HSCs were isolated and cultured from both Lrat-*Mpc2*^-/-^ and wild-type (WT) littermate mice. After 7 days in culture, there was a marked increase in the expression of several HSC activation markers in both groups, but the expression of these markers was substantially reduced in the Lrat-*Mpc2*^-/-^ HSC compared with WT cultures (Figure 1*E*). Collectively, our results demonstrate that genetic inhibition of the MPC reduces HSC activation *in vitro.*

### Lrat-*Mpc2*^-/-^ Mice are Protected From MASH-inducing Diet

**Figure 2:**
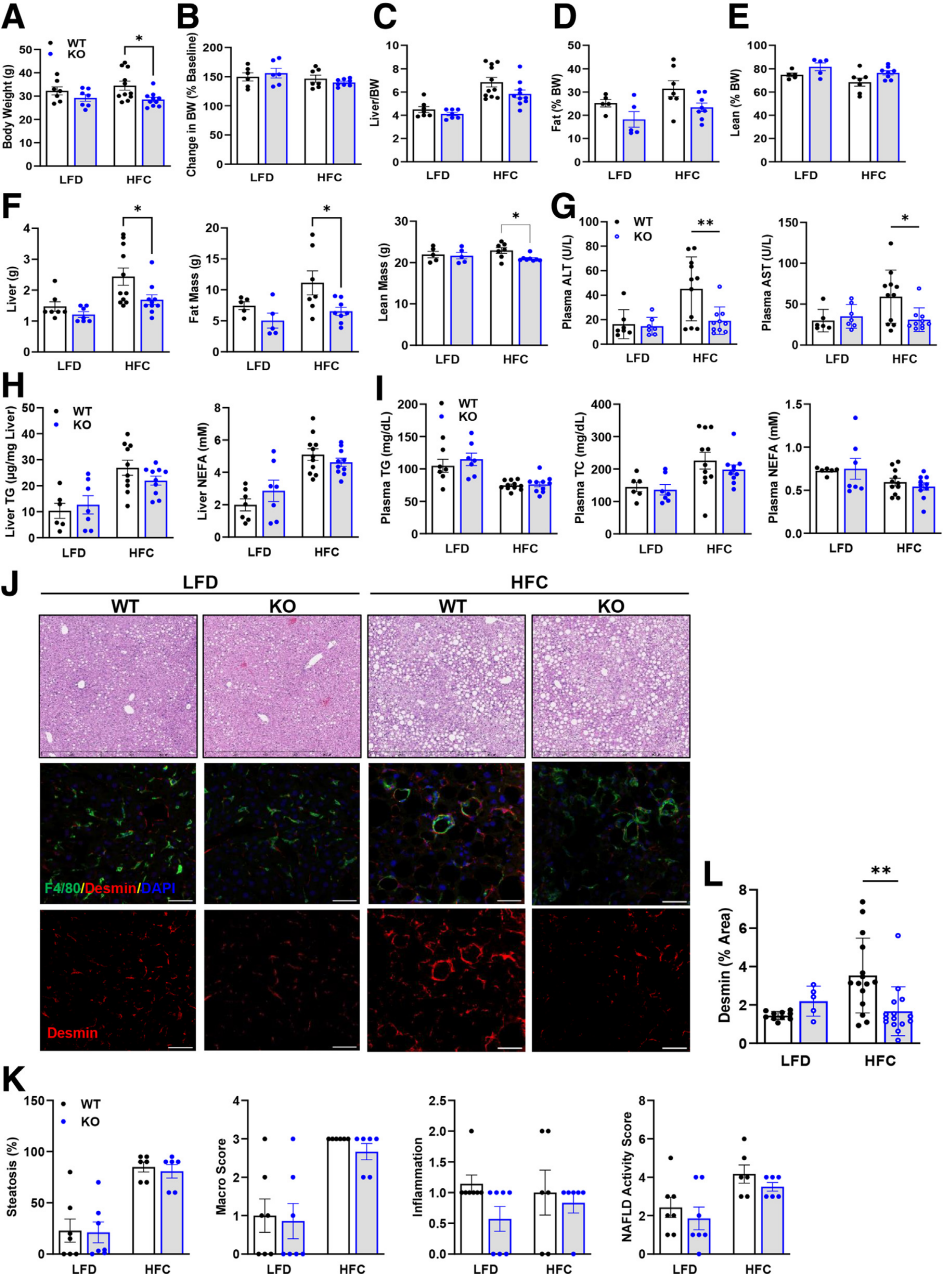
**Lrat-*Mpc2*^-/-^ mice are protected from MASH-inducing diet (HFC).** At about 8 weeks of age, littermate male WT and Lrat-Mpc2-/- (knockout) mice were placed on either a LFD or an HFC diet for a period of 12 weeks. (*A–B*) Terminal body weight and changes in body weight relative to the baseline. (*C*) Liver weight, measured at sacrifice, relative to terminal body weight. (*D–E*) body composition, determined by EchoMRI, expressed as mean ± SEM (n = 7–11/group). (*F*) Liver weight, fat mass, and lean mass measured at sacrifice. (*G*) Plasma levels of circulating transaminases ALT and AST collected at sacrifice. (*H, I*) Analysis of triglycerides (TG), total cholesterol (TC), and NEFA from liver (*H*) and plasma (*I*), expressed as mean ± SEM (n = 7–11/group). (*J*) Representative liver sections with H&E staining and immunofluorescence staining for hepatic stellate cells (Desmin), macrophages (F4/80), and nuclei (DAPI). (*K*) Histologic scoring of H&E-stained liver sections assessing steatosis, macrosteatosis, lobular inflammation, and NAFLD activity score expressed as mean ± SEM (n = 6–7/group). (*L*) Quantification of desmin staining.

**Figure 3:**
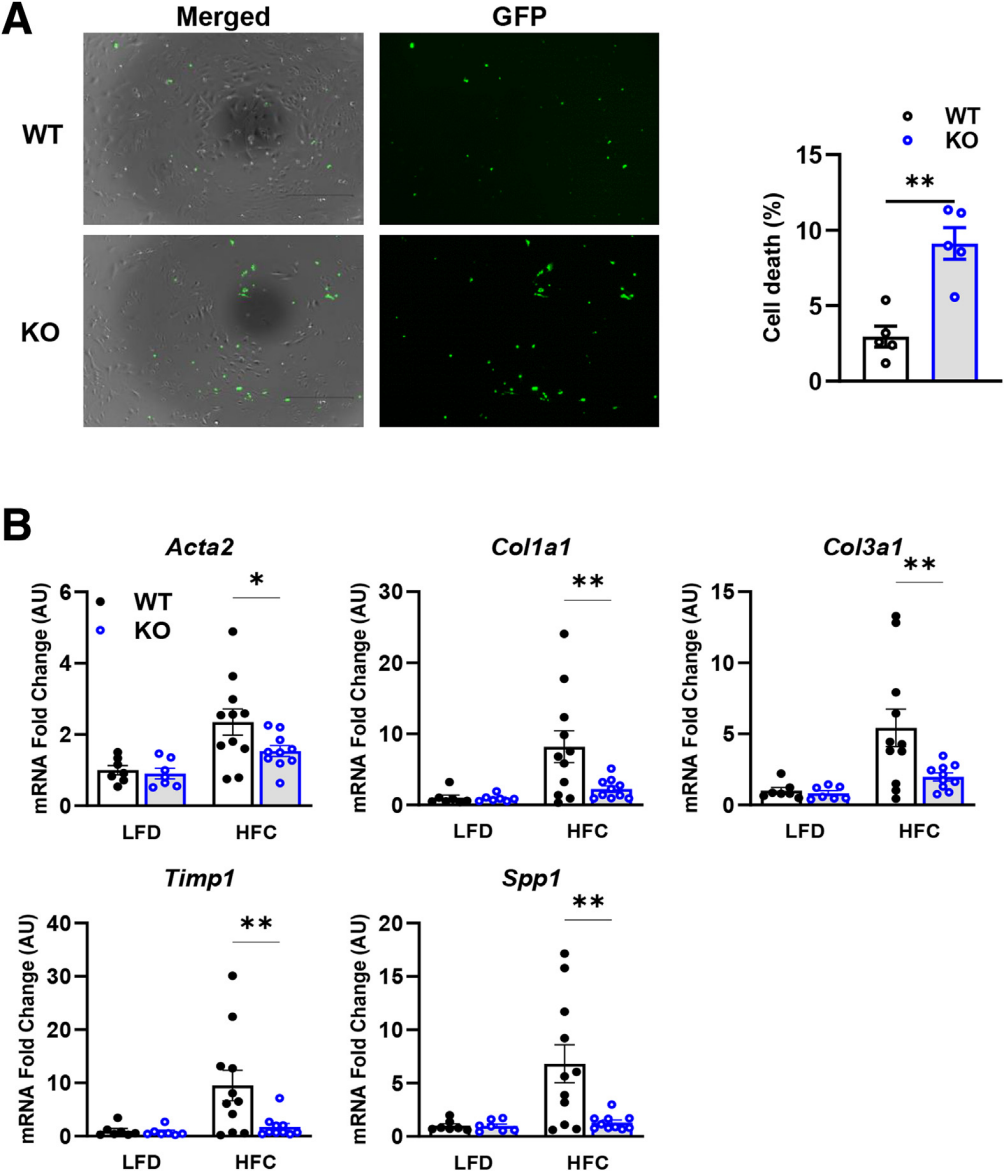
**Lrat-*MPC2*^-/-^ induced cell death and protected from MASH-inducing diet (HFC).** (*A*) Representative images indicating primary HSCs from knockout (KO) and WT mice stained using SYTOX green nucleotide stain with quantification. ∗∗*P* < .01. (*B*) Hepatic gene expression measured by RT-qPCR and expressed relative to WT LFD control group. All data expressed as mean ± SEM (n = 7–11/group). ∗*P* < .05; ∗∗*P*< .01 vs WT mice on the same diet as determined by 2-way ANOVA, followed by Sidak’s test for multiple comparisons.

Next, we sought to examine how MPC inhibition in stellate cells affects liver injury and HSC activation in vivo. Male Lrat-*Mpc2*^-/-^ and WT littermate mice were placed on either a low-fat diet (LFD) or a diet high in fat, fructose, and cholesterol (HFC) for a period of 12 weeks with a single dose of carbon tetrachloride after 4 weeks on the HFC diet. At the start of diet feeding and after 12 weeks of diet, Lrat-*Mpc2*^-/-^ mice had an average body weight that was slightly less than WT littermates, but both genotypes gained a similar amount of weight during dietary feeding (Figure 2*A–B*). Lrat-*Mpc2*^-/-^ mice on the HFC diet had a significant reduction in liver weight, fat mass, and lean mass, compared with WT mice; however, when normalized to total body weight, there was no difference in between groups (Figure 2*C–F*). Next, we assessed plasma transaminases, alanine transaminase (ALT) and aspartate aminotransferase (AST), as markers of liver injury and found that on the HFC diet Lrat-*Mpc2*^-/-^ mice had a significant reduction in ALT and AST levels compared with WT mice (Figure 2*G*). There were no changes in plasma or intrahepatic lipid concentrations (Figure 2*H–I*). Histologic examination of hematoxylin and eosin (H&E)-stained liver sections revealed a slight trend towards reduced macrosteatosis, inflammation, and nonalcoholic fatty liver disease (NAFLD) activity score, but these did not reach statistical significance (Figure 2*J–K*). In contrast, quantification of desmin staining of HFC fed mice demonstrated a significant decrease in the number of mature HSC in Lrat-*Mpc2*^-/-^ mice relative to WT mice, whereas HSC number was similar between genotypes on an LFD (Figure 2*J–L*). To evaluate the effects of MPC2 knockout on HSC cell death, primary HSC from Lrat-*Mpc2*^-/-^ mice were isolated, and cell death was evaluated by using SYTOX nucleic acid stain. Consistent with the observed decrease in HSC numbers in vivo, loss of MPC2 resulted in increased cell death in vitro (Figure 3*A*).

Lastly, we measured hepatic gene expression and found that Lrat-*Mpc2*^-/-^ mice had significantly decreased expression of several markers of HSC activation, including multiple collagen isoforms, *Acta2*, *Timp1*, and *Spp1* (Figure 3*B*). Taken together, our results suggest that *Mpc2* deletion in HSCs reduces the expansion and activation of these cells and protects from MASH development in mice fed a high-fat diet (HFD).

### Lrat-*Mpc2*^-/-^ Mice are Protected From MASH on a Choline-deficient L-amino Acid-defined Diet

**Figure 4:**
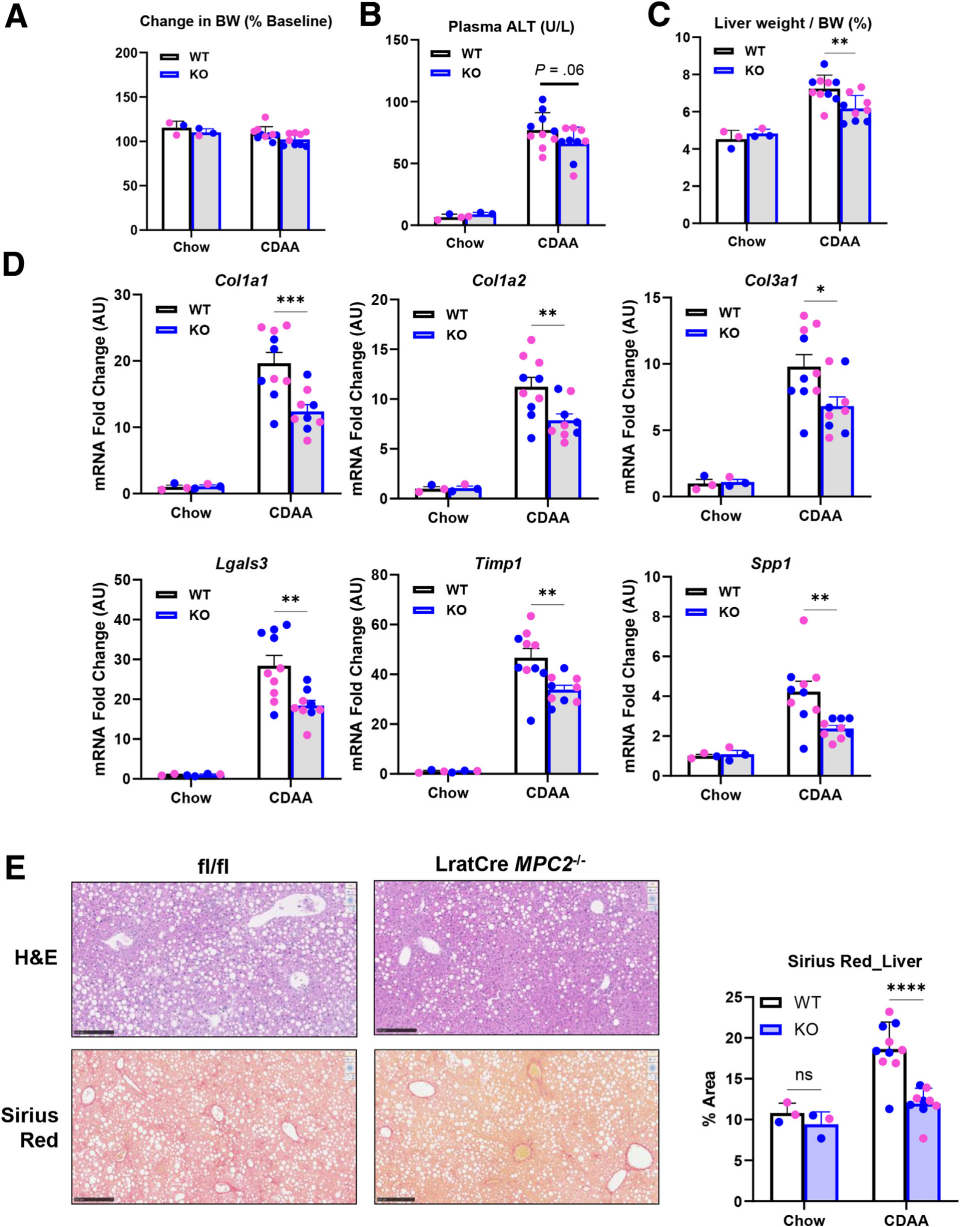
**MPC deletion in hepatic stellate cells protects mice from MASH-inducing diet (CDAA).** At about 10 weeks of age, male (*blue data points*) and female (*pink data points*) Lrat-*Mpc2*^-/-^ knockout (KO) and WT littermates received a CDAA diet for a period of 10 weeks. (*A*) Body weight change in experimental period reported as percentage relative to initial body weight. (*B*) Plasma levels of ALT at sacrifice. (*C*) Liver weight measured at sacrifice normalized to final body weight. (*D*) Hepatic gene expression measured by RT-qPCR and expressed relative to WT chow control group. All data expressed as mean ± SEM (n = 3 [chow] or 10 [CDAA]/group). ∗*P*< .05; ∗∗*P*< .01; ∗∗∗*P*< .001 vs WT mice on the same diet as determined by 2-way ANOVA, followed by Sidak’s test for multiple comparisons. (*E*) Representative liver sections with H&E and Sirius red staining in knockout (KO) and WT mice fed a CDAA diet with quantification ∗∗∗∗*P* < .001.

We also examined the effects of feeding a choline-deficient defined amino acid (CDAA) diet with higher fat content (45 kcal% fat) in male and female WT and Lrat-*Mpc2*^-/-^ mice. There was no effect of diet or genotype on weight gained during the trial (Figure 4*A*). The CDAA diet provoked a marked increase in plasma ALT, and Lrat-*Mpc2*^-/-^ mice tended to have lower ALT levels compared with WT mice on a CDAA diet (Figure 4*B*). Lrat-*Mpc2*^-/-^ mice also exhibited a significantly reduced liver weight to body weight ratio on a CDAA diet (Figure 4*C*). The expression of markers of HSC activation, which was markedly increased by a CDAA diet, was significantly blunted in Lrat-*Mpc2*^-/-^ mice (Figure 4*D*). H&E staining suggested that steatosis was not different between liver tissue of Lrat-*Mpc2*^-/-^ and WT mice. However, Sirius Red staining detected less hepatic fibrosis in Lrat-*Mpc2*^-/-^ fed a CDAA diet compared with WT littermates (Figure 4*E*). Collectively, these data demonstrate that stellate cell-specific disruption of the MPC blunts HSC activation in multiple models of diet-induced MASH in mice.

### Transcriptional Responses to MPC Deletion in HSCs

**Figure 5:**
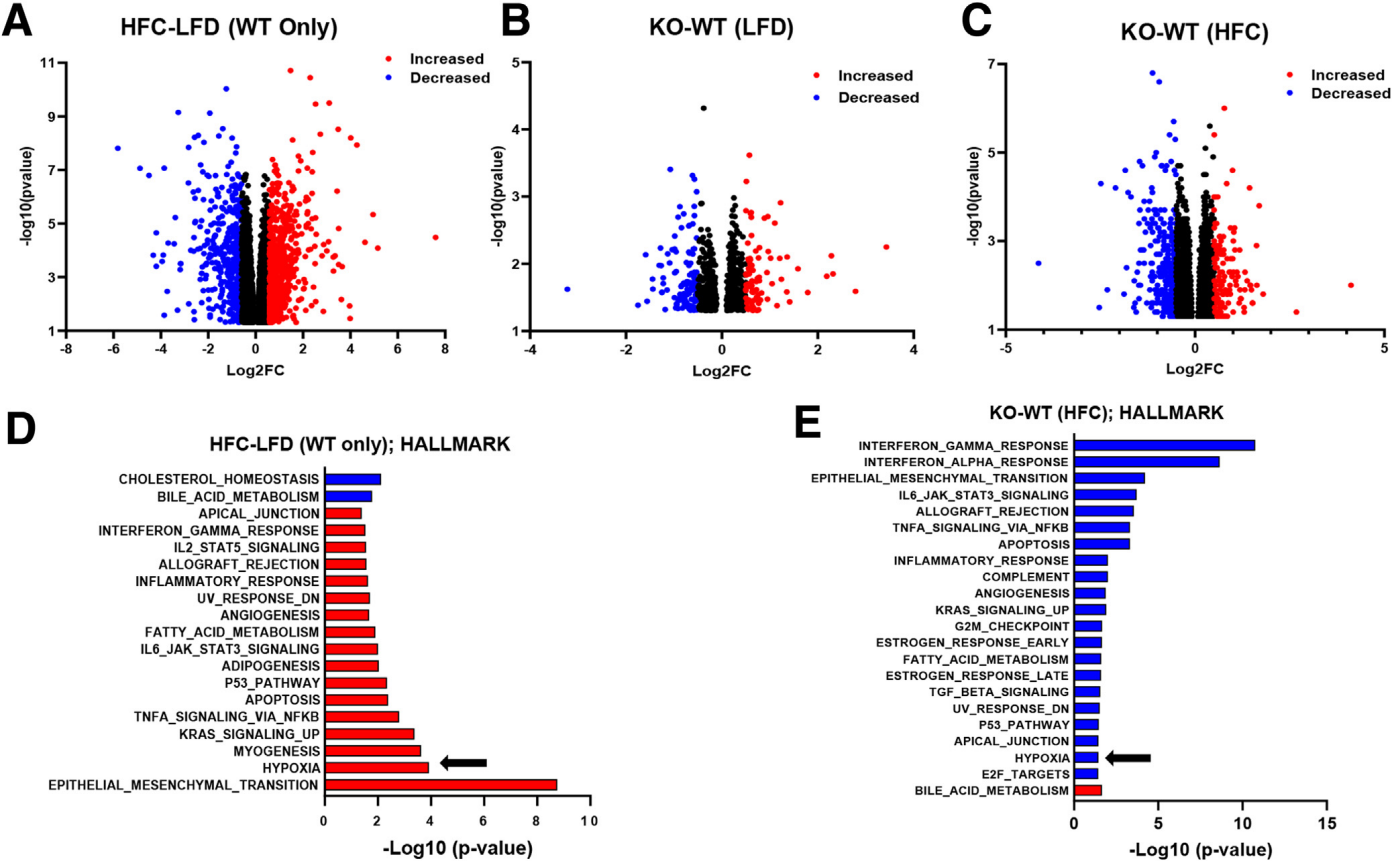
**Lrat-*Mpc2*^-/-^ mice are protected from MASH-inducing diets (HFC).** (*A–C***)** Volcano plots of differentially expressed genes with *P*< .05 comparing (*A*) HFC vs LFD in WT mice, (*B*) knockout (KO) vs WT mice on LFD, and (*C*) KO vs WT mice on HFC diet. DEGs with Log fold change (LogFC) less than −0.5, or greater than 0.5, were highlighted in either *blue* or *red*, respectively. (*D–E*) GSEA of perturbations in Hallmark gene set collections when comparing (*D*) HFC vs LFD in WT mice and (*E*) KO vs WT mice on HFC diet. The arrows highlight hypoxia signaling pathways. Differential expression analysis was then performed to analyze for differences between conditions. RNA-seq was performed on liver tissue from WT and Lrat-*Mpc2*-/- knockout (KO) mice on either a LFD or HFC diet (n = 5/group).

To gain a better understanding of the differences in the hepatic transcriptome between Lrat-*Mpc2*^-/-^ and WT mice, we performed bulk RNA sequencing (RNAseq) analysis on livers from both genotypes on LFD and HFC diets. Analysis of differentially expressed genes (DEGs) revealed that WT mice exhibited robust changes in the transcriptome in response to HFC feeding, with the expression of 559 genes being decreased and 960 genes being increased compared with LFD comparators (Figure 5*A*). On a LFD, the expression of 214 genes significantly changed (123 decreased and 91 increased) in Lrat-*Mpc2*^-/-^ mice compared with WT mice (Figure 5*B*). Comparison within the HFC groups revealed reduced expression of 343 genes, whereas the expression of 133 genes was increased in Lrat-*Mpc2*^-/-^ livers compared with WT mice (Figure 5*C*). As might be predicted, gene set enrichment analysis (GSEA) comparing LFD with HFC groups of WT mice revealed a marked activation of pathways, including epithelial to mesenchymal transition, inflammation and immune responses, and apoptosis in response to HFC (Figure 5*D*). In contrast, a decrease in the expression of pathways involved in immune responses, epithelial to mesenchymal transition, and apoptosis in Lrat-*Mpc2*^-/-^ relative to WT mice in HFC-fed mice was observed (Figure 5*E*). Additionally, pathways associated with fatty acid metabolism were decreased, whereas the bile acid metabolism pathway was increased in Lrat-*Mpc2*^-/-^ mice compared with WT mice. Collectively, these results indicate that stellate cell-specific deletion of *Mpc2* results in a shift in the global hepatic transcriptome that is associated with a decreased immune response and altered metabolism.

**Figure 6:**
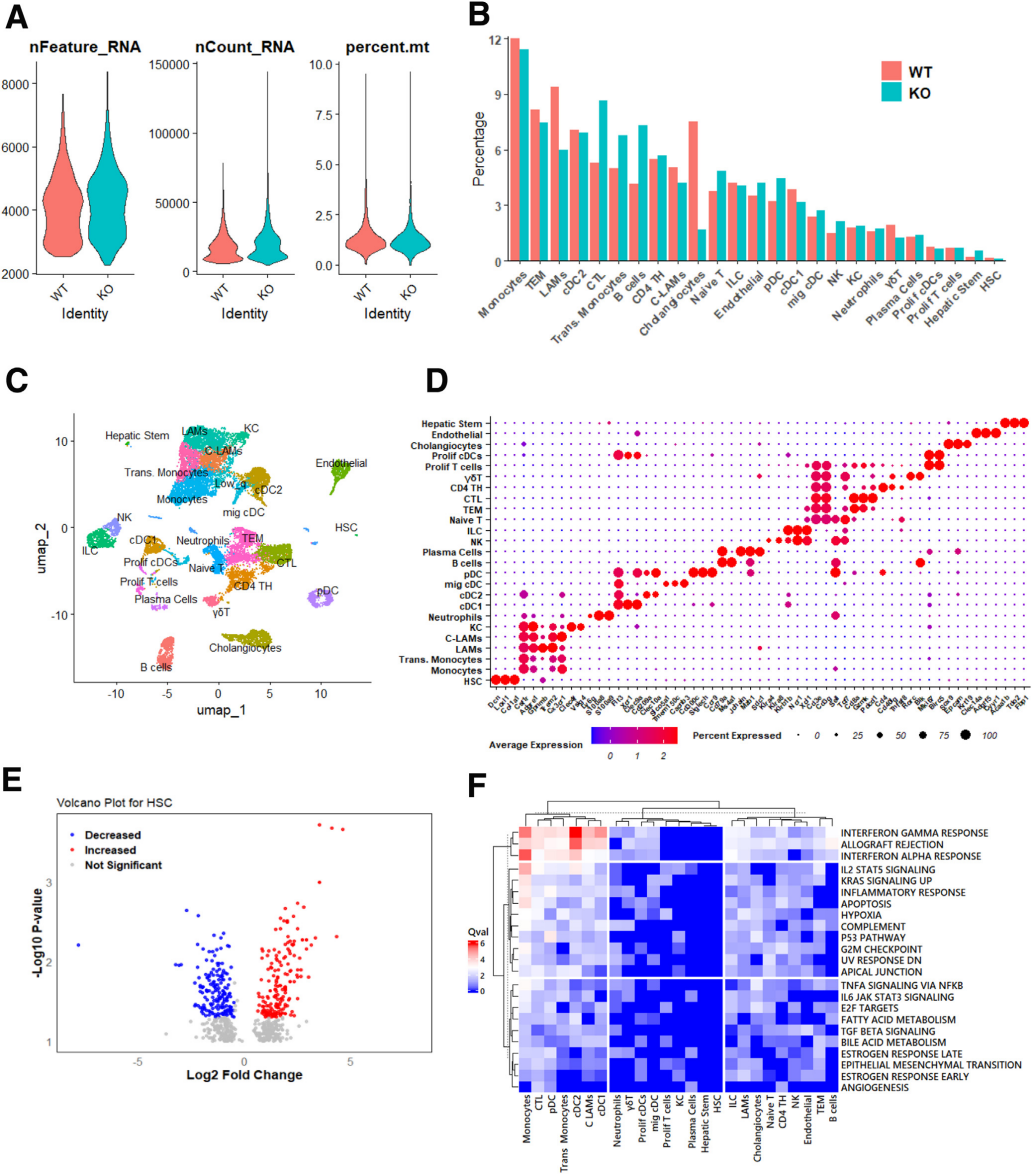
**Lrat-Mpc2-/- mice are protected from MASH-inducing diets (CDAA).** (*A*) Violin plots of number of genes detected per cell, number of UMIs per cell, and proportion of cell reads from mitochondrial genes. **(***B*) Bar graph of annotated cell clusters expressed as percent of all cells in each respective genotype. (*C*) Uniform manifold approximation and projection (UMAP) of non-parenchymal cells from WT and knockout (KO) mice after CDAA diet-feeding for 10 to 12 weeks. (*D*) Dot plot of cell type specific markers for each cell cluster used for annotation. (*E*) Volcano plot of DEGs in the HSC cluster when comparing KO vs WT. Genes that had *P* < .05 were highlighted in either *red* (Log2FC >0.5) or *blue* (Log2FC <0.5) in KO vs WT. (*F*) Heatmap representation of highly altered Hallmark gene set pathways among all clusters when comparing KO vs WT using SCPA, expressed by the Qval (Q value statistic), which represents the size of distribution for change within a given pathway. cDC1 (conventional type 1 dendritic cell), cDC2 (conventional type 2 dendritic cell), CD4 TH (CD4 t helper), C-LAMs, CTL, γδT, hepatic stem (hepatic stem/progenitor cells), HSCs, ILC, KC, LAMs, Low q (cells with low RNA quality [high mitochondrial genes, low RNA count]), mig cDC (migratory conventional dendritic cell), NK, pDC (plasmacytoid dendritic cell), prolif cDCs (proliferating conventional dendritic cells), prolif T cells (proliferation T cells), trans monocytes (transitioning monocytes), T_EM_.

Because the bulk RNAseq detected decreased immune responses, possibly reflecting alterations in immune cell infiltration or activation, we conducted single-cell RNA sequencing (scRNAseq) on the non-parenchymal cell fraction from livers of mice fed a CDAA diet for 10 to 12 weeks using fluorescence-activated cell sorting after enzymatic digestion. Following quality control filtering, we obtained 10,210 and 8239 cells from WT and Lrat-*Mpc2*^-/-^ mice, respectively (Figure 6*A–B*). Unsupervised clustering revealed several distinct cell types that were annotated using cell-specific markers (Figure 5*C–D*). Most cells recovered were immune cells, but we also observed populations of cholangiocytes (*Sox9*, *Epcam*, *Krt19*), endothelial cells (*Clec14a*, *Adgrf5*, *Cyyr1*), and HSCs (*Dcn*, *Loxl1*, *Col1a1*). Myeloid cell clusters included monocytes/macrophages (*Csfr1*, *Adgre1*), type 1 conventional dendritic cells (cDC1; *Flt3*, *Xcr1*, *Clec9a*), type 2 conventional dendritic cells (cDC2; *Flt3*, *Cd209a*, *Clec10a*), migratory dendritic cells (mig cDC; *Slco5a1*, *Tmem150c*, *Cacnb3*), and plasmacytoid dendritic cells (pDC; *Flt3*, *Cd300c*, *Siglech, Ccr9*). Macrophage clusters consisted of Kupffer cells (KC; *Clec4f*, *Vsig4*), lipid-associated macrophages (LAMs; *Gpnmb*, *Trem2*), and crown-like LAMs (C-LAMs; high *Cx3cr1*, *Trem2*). We observed several T cells clusters (*Cd3e*, *Cd3g*) including naïve T cells (*Sell*, *Tcf7*), T effector memory cells (T_EM_; *Cd8a*, *Cx3cr1*, *Gzmk*), cytotoxic T lymphocytes (CTL; *Cd8a*, *Gzmk*, *Pdcd1*), CD4 T helpers (CD4 T_H_; *Cd4*, *Cd40lg*, *Tnfsf8*), and gamma delta T cells (γδTs; *Rorc*, *Blk*). Other lymphocytes included B cells (*Ms4a1*, *Cd79a*), plasma cells (*Jchain*, *Sdc1*), natural killer cells (NKs; *Klra4*, *Klra8*), and innate lymphoid cells (ILCs; *Ncr1*, *Xcl1*). A cluster of low quality (Low_q) containing high mitochondrial DNA and low RNA count was excluded from downstream analyses. Although all cell types were represented in both genotypes, there were several notable differences in the percent of certain cell types (Figure 6*E*). Specifically, Lrat-*Mpc2*^-/-^ mice had lower proportions of cholangiocytes (1.7% vs 7.5%) and LAMs (6.0% vs 9.4%), compared with WT mice. In contrast, there were higher percentages of CTLs (8.7% vs 5.3%) and B cells (7.3% vs 4.2%) in Lrat-*Mpc2*^-/-^ relative to WT mice. Unfortunately, there was a low number of HSCs in both the WT (17 cells) and Lrat-*Mpc2*^-/-^ (9 cells) mice, and we were unable to perform meaningful downstream analyses.

### Effects of HSC MPC deletion on Other Hepatic Non-parenchymal Cells

To assess the transcriptional responses that are secondary to HSCs, we performed a single-cell pathway analysis (SCPA)^24^ to characterize differential changes in pathway activity across all our cell types. The SCPA defines pathway activity as a change in multivariate distribution of a given pathway across different populations and conditions, which is represented statistically as a q value. SCPA analysis of Hallmark gene sets revealed alterations in many of the same gene sets found to be perturbed in our bulk RNAseq data, including interferon responses, JAK-STAT signaling, apoptosis, and hypoxia, as determined by a high q value statistic that indicates the degree of change for a particular pathway (Figure 6*F*). As with our bulk RNAseq data, INTERFERON RESPONSES had the highest degree of change and was most prominent in dendritic cells (cDC1, cDC2, pDC), CTLs, monocytes, and macrophages (C-LAMs). In contrast to our bulk RNAseq data, there was an increase in the interferon responses in most cell types, except for a decrease in the cholangiocyte clusters (data not shown). Similar trends were also observed for the IL6-JAK-STAT3 pathway. Both APOPTOSIS and HYPOXIA gene sets were highly altered in monocytes and LAMs/C-LAMs and were decreased in most cell types apart from cholangiocytes. Notably, we saw no changes in the HSC cluster, likely due to a low number of HSC cells analyzed.

**Figure 7:**
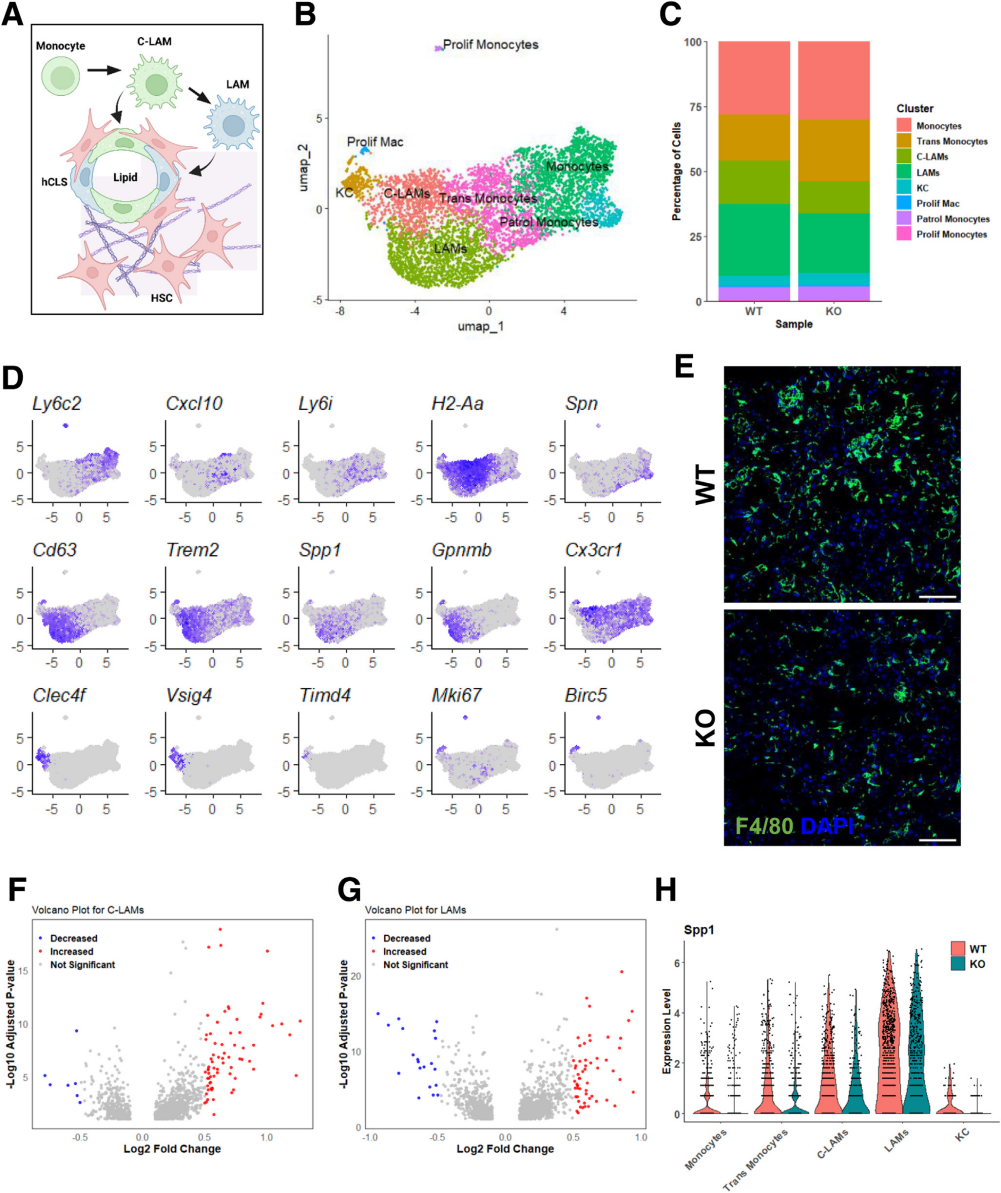
**Lrat-*Mpc2*^-/-^ mice have altered transcriptional responses in myeloid cells.** (*A*) Schematic depicting interaction between macrophages and HSCs. In response to chronic steatotic liver disease, circulating monocytes enter the liver and differentiate into distinct populations of macrophages that localize in areas of lipid accumulation and fibrosis, forming hepatic crown-like structures, that are associated with increased stellate cells and fibrosis. (*B*) Uniform Manifold Approximation and Projection (UMAP) of the monocyte and macrophage subsets. (*C*) Bar graph of monocyte/macrophage subset clusters expressed as percent within each respective genotype. (*D*) UMAP representation of previously established markers that were used to annotate monocyte/macrophage clusters. (*E*) Representative immunofluorescence staining of liver sections for macrophages (F4/80) (Scale bar = 100 μm). (*F–G*) Volcano plot of DEGs in the C-LAM and LAM clusters, respectively, when comparing KO vs WT. Genes that had *P* < .05 were highlighted in either *red* (Log2FC >0.5) or *blue* (Log2FC <0.5) in knockout (KO) vs WT. (*H*) Violin plots of *Spp1* expression in WT and KO monocyte/macrophage subsets.

Because monocytes/macrophages represent a large portion of our scRNAseq dataset, and HSCs have been shown to influence monocyte/macrophage phenotypes during MASH (Figure 7*A*),^25^ we took these clusters and analyzed them further. We found 8 different clusters characterized by cell-specific markers including monocytes (*Ly6c2*, low expression of H2-Aa), transitioning monocytes (*Cxcl10*, *Ly6i*, *H2-Aa*), patrolling monocytes (*Spn*, *Eno3*), LAMs (*Cd63, Trem2, Spp1, Gpnmb*), C-LAMs (high Cx3cr1 expression, *Cd63, Gpnmb*), KCs (*Clec4f, Vsig4*), and proliferating cells (*Mki67, Birc5*) (Figure 7*B, D*). In the KC cluster, there was very low expression of *Timd4*, suggesting that most of the KCs were monocyte-derived, which is consistent with prior studies of KC in MASH.^26^ Similar to the analysis of all cell clusters, we found that Lrat-*Mpc2*^-/-^ mice had a decrease in the percent of LAMs and C-LAMs, while there was an increase in transitioning monocytes (Figure 7*C*). Additionally, liver sections stained with the macrophage marker F4/80 indicated a trend towards reduced macrophage accumulation in Lrat-*Mpc2*^-/-^ mice relative to WT mice (Figure 7*E*). Examination of gene expression in the C-LAM population revealed 7 down-regulated and 71 upregulated genes (*P* < .05; Log2FC ≤ −0.5) in Lrat-*Mpc2*^-/-^ relative to WT mice (Figure 7*F*). In the LAM population, there were 20 genes downregulated and 56 upregulated (*P* < .05; Log2FC ≥ 0.5) in Lrat-*Mpc2*^-/-^ compared with WT mice (Figure 7*G*). Interestingly, expression of *Spp1*, the gene encoding osteopontin and associated with more advanced liver disease progression,^27^ seemed to be decreased in multiple clusters including monocytes, transitioning monocytes, C-LAMs, and LAMs in Lrat-*Mpc2*^-/-^ mice compared with WT mice (Figure 7*H*). Diminished expression of *Spp1* was also noted in total liver RNA of Lrat-*Mpc2*^-/-^ mice on MASH-inducing diets (Figures 3*B* and 4*D*). Overall, HSC-specific MPC inhibition altered intrahepatic macrophage compositions, which are important regulators of MASH progression and development of fibrosis.

### Metabolic Effects of MPC Inhibition in HSCs

**Figure 8:**
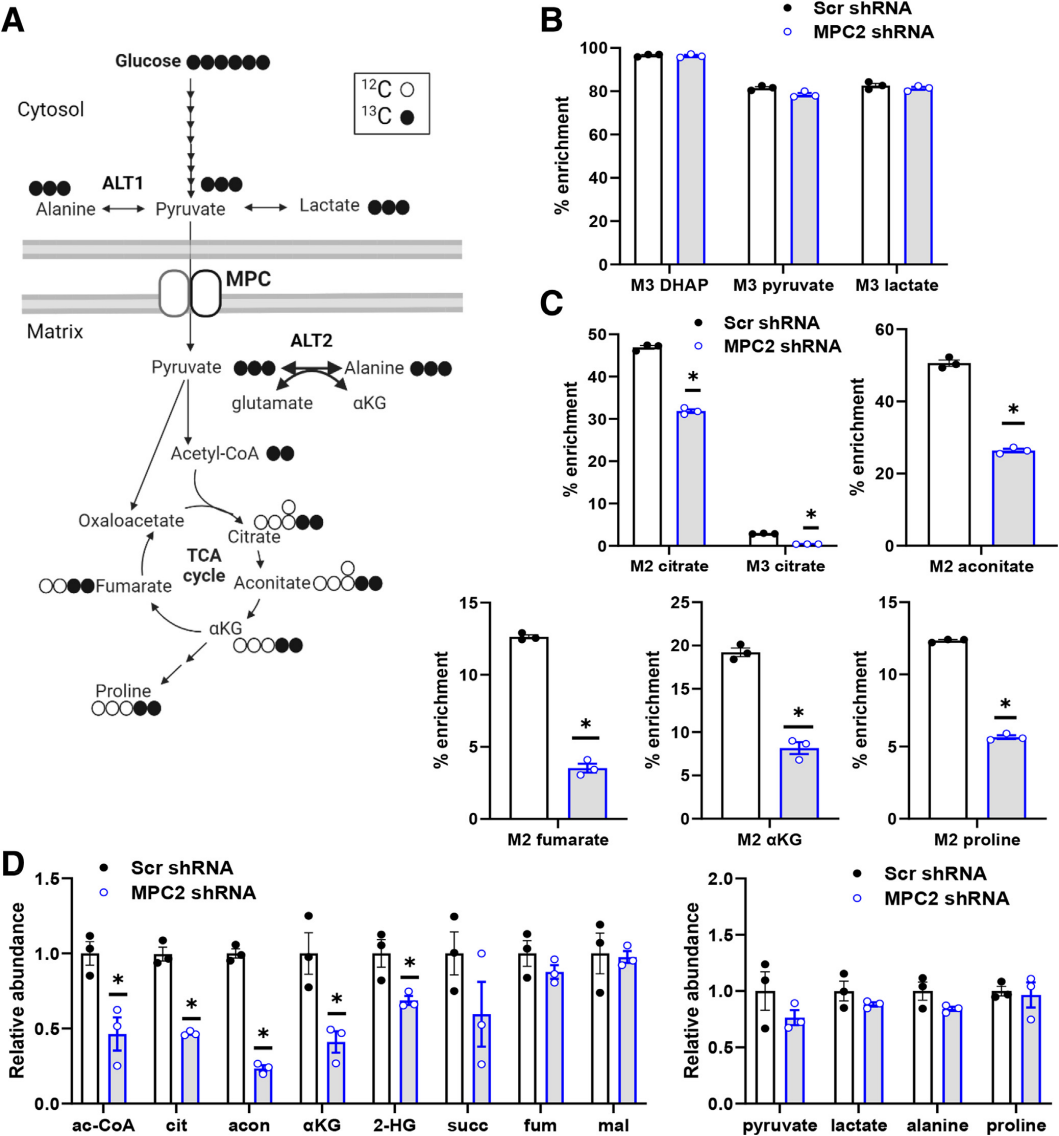
**MPC suppression markedly reduces several TCA cycle intermediates in LX2 cells treated with uniformly labeled ^13^C-glucose.** (*A*) Schematic of glucose metabolism and the TCA cycle tracing the route of incorporation of ^13^C-glucose. (*B–C*) TGF-β-1 (5 ng/mL) was added into media and LX2 cells expressing either Scr control shRNA or shRNA against MPC2. Cells were then cultured overnight in the presence of uniformly labeled ^13^C-glucose. (*B*) Incorporation of ^13^C-glucose into glycolytic intermediates. (*C*) Incorporation of ^13^C-glucose into TCA cycle intermediates and amino acids; M2, M3, indicate 2 or 3 carbon atoms are derived from labeled glucose, respectively. (*D*) Relative abundance of intermediates in cells expressing MPC2 shRNA or Scr control shRNA. Data are expressed as mean ± SEM (n = 3) from a representative experiment. ∗*P* < .05 vs Scr shRNA as determined by unpaired Student *t*-test. Schematics created with BioRender.com

To characterize LX2 cell metabolism and the effects of MPC inhibition on relevant metabolic pathways, we administered uniformly labeled ^13^C-glucose to control or MPC2 knockdown cells and then evaluated incorporation of ^13^C into various metabolites (Figure 8*A*). Consistent with high rates of glycolysis in LX2 cells,^28^ ^13^C-glucose was abundantly incorporated into glycolytic intermediates including DHAP, pyruvate, and lactate, and this was not affected by MPC2 suppression (Figure 8*B*). In contrast, MPC2 shRNA markedly reduced the incorporation of ^13^C into TCA cycle intermediates (citrate, aconitate, fumarate, and αKG) that require mitochondrial import of pyruvate (Figure 8*A*). Comparison of M2- vs M3-citrate enrichment indicated that the majority of pyruvate was decarboxylated by the pyruvate dehydrogenase complex rather than carboxylated by pyruvate carboxylase. Proline is a critical constituent of extracellular matrix as a component of mature collagen,^29^ and roughly 10% of this amino acid was enriched with ^13^C-glucose (Figure 8*C*). Suppression of MPC activity markedly reduced incorporation of ^13^C-glucose into proline but did not affect the total abundance of this amino acid (Figure 8*D*), suggesting compensation by other metabolic pathways such as glutaminolysis.

MPC2 suppression markedly reduced the total abundance of several TCA cycle intermediates, including acetyl-CoA, citrate, aconitate, αKG, and 2-hydroxyglutarate (2-HG) (Figure 8*D*), which is consistent with the critical role of mitochondrial metabolism of glucose (as pyruvate) in generating these intermediates (Figure 8*A*). MPC shRNA did not affect the pool size of glycolytic end products including pyruvate, lactate, and alanine (Figure 8*D*). The pool sizes of all measured intermediates can be found in Supplementary Table 1. Collectively, these findings suggest that MPC knockdown alters the fate of glucose in LX2 cells and leads to depletion of TCA cycle intermediates, which may be critical for HSC activation.

### Effects of TCA Cycle Depletion on HSC Activation

**Figure 9:**
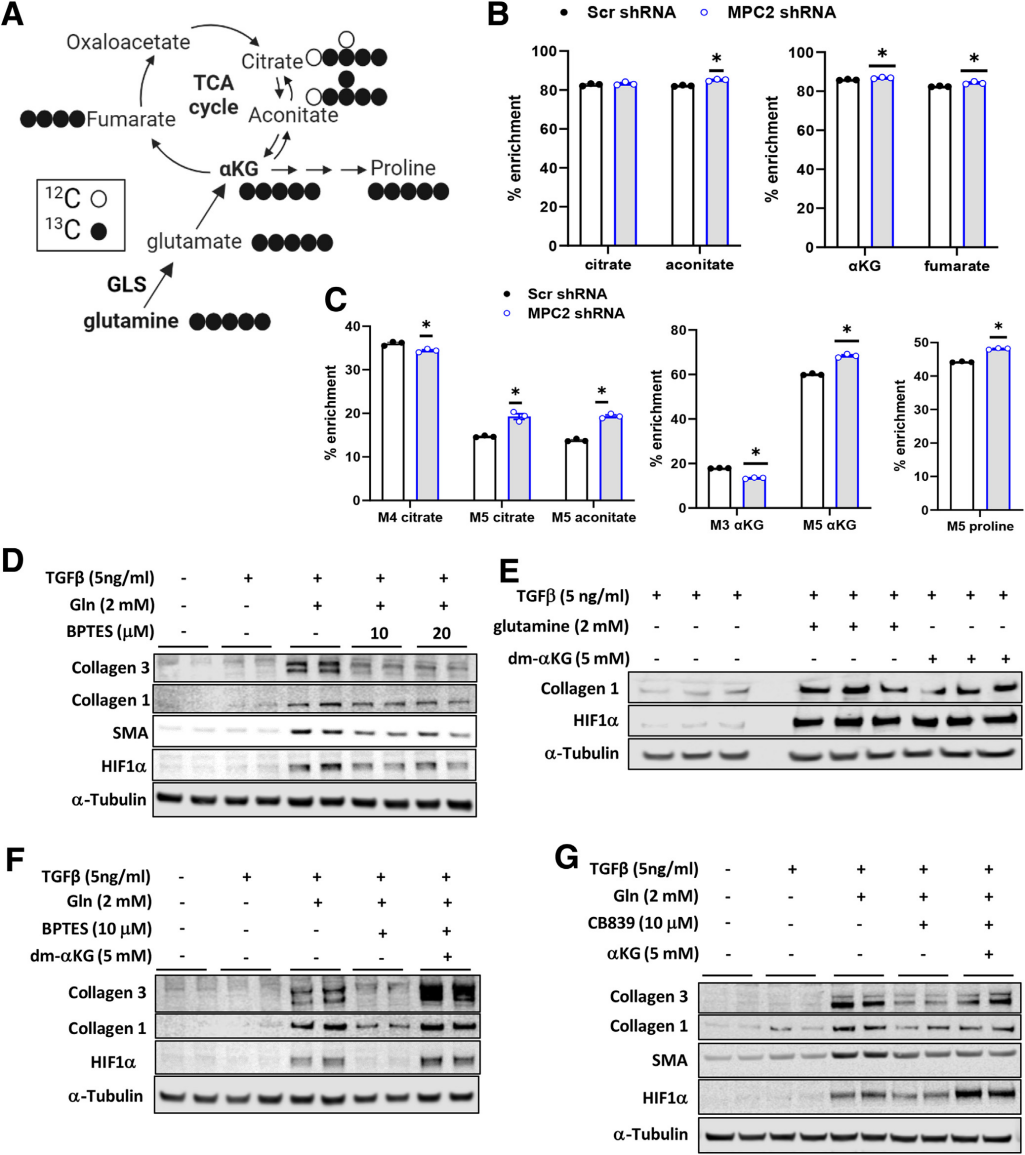
**MPC suppression markedly reduces several TCA cycle intermediates in LX2 cells treated with uniformly labeled ^13^C-glutamine.** (*A*) The schematic depicts glutaminolysis and the TCA cycle and traces the incorporation of ^13^C-glutamine. (*B–C*) Scr control or MPC2 shRNA expressing LX2 cells were treated with TGF-β-1 (5 ng/mL) and cultured overnight in the presence of uniformly-labeled ^13^C-glutamine. Enrichments of ^13^C-glutamine into TCA cycle intermediates and amino acids are shown. Data are expressed as mean ± SEM (n = 3) from a representative experiment. ∗*P* < .05 vs Scr shRNA as determined by unpaired Student *t*-test. (*D*) Western blots for collagen 3, collagen 1, SMA, HIF1α, and α-Tubulin in LX2 cells treated with or without TGF-β-1 (5 ng/mL), glutamine (Gln, 2 mM), and in the presence or absence of the glutaminase inhibitor BPTES (10 or 20 μM). **(***E*) Western blot images for collagen 1, HIF1α, and α-Tubulin in LX2 cells treated with or without glutamine (Gln, 2 mM) or dm-αKG (5 mM) compared with TGF-β-1 (5 ng/mL) treated cells. (*F–G*) Protein abundance of the indicated proteins in LX2 cells treated with or without TGF-β-1 (5 ng/mL), glutamine (Gln, 2 mM), and (*F*) BPTES (10 μM) or (*G*) CB839. Cells in the last lane were supplemented with dm-αKG (5 mM). (*H*) Western blot images for collagen 1, SMA, HIF1α, and α-Tubulin in LX2 cells treated with or without TGF-β-1 (5 ng/mL) and HIF1α inhibitor VI (10 or 20 μM).

We also performed tracing studies with uniformly labeled ^13^C-glutamine in control and shMPC2 LX2 cells (Figure 9*A*). Total enrichment of ^13^C-glutamine in some TCA cycle intermediates and proline was slightly increased by MPC2 knockdown (Figure 9*B*), which is consistent with a compensatory increase in glutaminolysis. Interestingly, however, oxidative metabolism of glutamine, as reflected by the enrichments in M4-citrate and M3-αKG, was decreased, whereas the reductive metabolism of glutamine (M5-citrate and M5-aconitate) was increased (Figure 9*C*). Consistent with prior work,^13^^,^^14^^,^^17^ inhibition of glutaminolysis was also sufficient to prevent LX2 cell activation in response to glutamine stimulation (Figure 9*D*).

Glutamine enters the TCA cycle as αKG, and metabolomic analyses demonstrated that αKG, an important TCA cycle intermediate and signaling molecule, was depleted in cells with MPC knockdown (Figure 8*D*). We therefore tested the sufficiency of αKG to stimulate collagen expression by depriving cells of glutamine and then providing them with a cell permeable αKG analog, dimethyl-αKG (dm-αKG). Interestingly, administration of dm-αKG to LX2 cells enhanced collagen protein expression even in the absence of glutamine (Figure 9*E*). Moreover, the effects of inhibiting glutaminolysis with BPTES (Figure 9*F*) or CB839 (Figure 9*G*) on HSC activation could be overcome by adding back dm-αKG. These findings support the concept that suppressing pyruvate or glutamine metabolism in HSCs is sufficient to impair the ability of LX2 cells to initiate the activation program.

### Regulation of HIF1α by Intermediary Metabolism

HIF1α is a transcription factor that plays important roles in HSC activation^30^ by directly regulating the expression of several genes important for HSC activation, including *COL1A1*.^31^^,^^32^ HIF1α protein stability is regulated by prolyl hydroxylase (PHD) enzymes, which hydroxylate HIF1α leading to its degradation. The reaction catalyzed by PHDs uses αKG as a substrate, converting it to succinate. In many types of cells, succinate suppresses PHD activity, leading to increased HIF1α abundance, whereas αKG administration enhanced PHD activity and reduced HIF1α.^33^, ^34^, ^35^ However, there is also evidence that αKG supplementation led to HIF1α stabilization in lung fibroblasts.^36^ To determine whether and how HIF1α was affected by metabolite availability in LX2 cells, we measured HIF1α protein abundance in the presence or absence of dm-αKG or glutamine. The addition of either dm-αKG or glutamine markedly increased the abundance of HIF1α (Figure 9*E*). Similarly, inhibiting glutaminolysis with BPTES (Figure 9*D, F*) or CB839 (Figure 9*G*) reduced HIF1α protein abundance, and this could be overcome by adding back dm-αKG.

### MPC Suppression Leads to Diminished Activation of HIF1α

**Figure 10:**
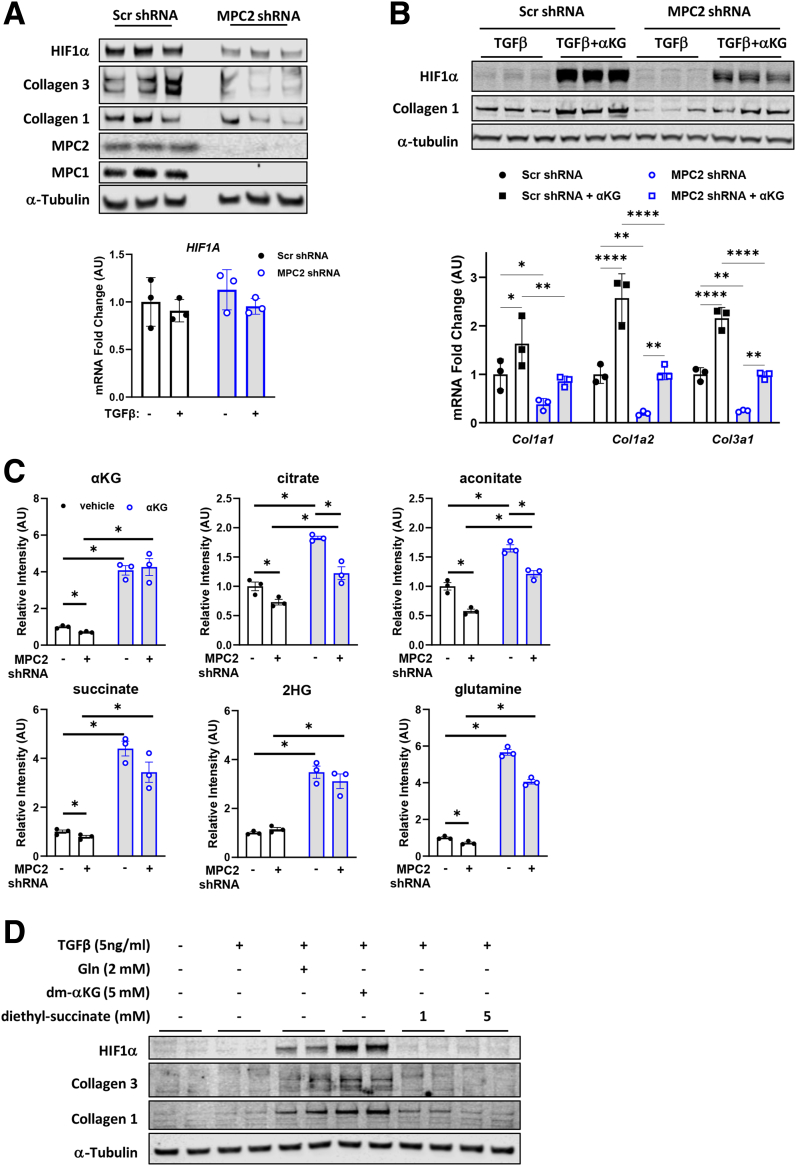
**MPC deficiency leads to impaired HIF1α activation by a metabolic mechanism.** (*A*) Western blot images for HIF1α, collagen 3, collagen 1, MPC2, MPC1, and α-Tubulin in LX2 cells expressing shRNA against MPC2. Cells treated with TGF-β-1 (5 ng/mL). Graph below depicts *HIF1A* mRNA expression in cells treated similarly. (*B*) Western blot images for collagen 1, HIF1α, and α-Tubulin in lysates from LX2 cells expressing Scr or MPC2 shRNA and treated with TGF-β-1 (5 ng/mL) with or without dm-αKG (5 mM). Graph below depicts mRNA expression of collagen genes in LX2 cells treated in the same way. (*C*) Relative intensity of TCA cycle intermediates in LX2 cells expressing Scr or MPC2 shRNA treated with TGF-β-1 (5 ng/mL) with or without dm-αKG (5 mM); Data are expressed as mean ± SEM (n = 3) from a representative experiment. ∗*P* < .0001 vs Scr shRNA as determined by 2-way ANOVA followed by Tukey’s post-hoc test. (*D*) Western blot images for HIF1α, collagen 1, collagen 3, and α-Tubulin in LX2 cells treated with or without TGF-β-1 (5 ng/mL), glutamine (Gln, 2 mM), dm-αKG (5 mM), or diethyl-succinate (1 or 5 mM).

Next, we examined the effects of MPC inhibition on HIF1α abundance and found that, compared with scrambled control expressing cells, shMPC2 reduced HIF1α abundance concordant with reduced collagen protein abundance in TGFβ-stimulated LX2 cells (Figure 10*A*). *HIF1A* mRNA was not affected by shMPC2, consistent with a post-transcriptional mechanism. Providing cells with dm-αKG partially overcame the effects of shMPC2 on HIF1α and collagen 1 protein abundance and collagen mRNA expression (Figure 10*B*).

**Figure 11:**
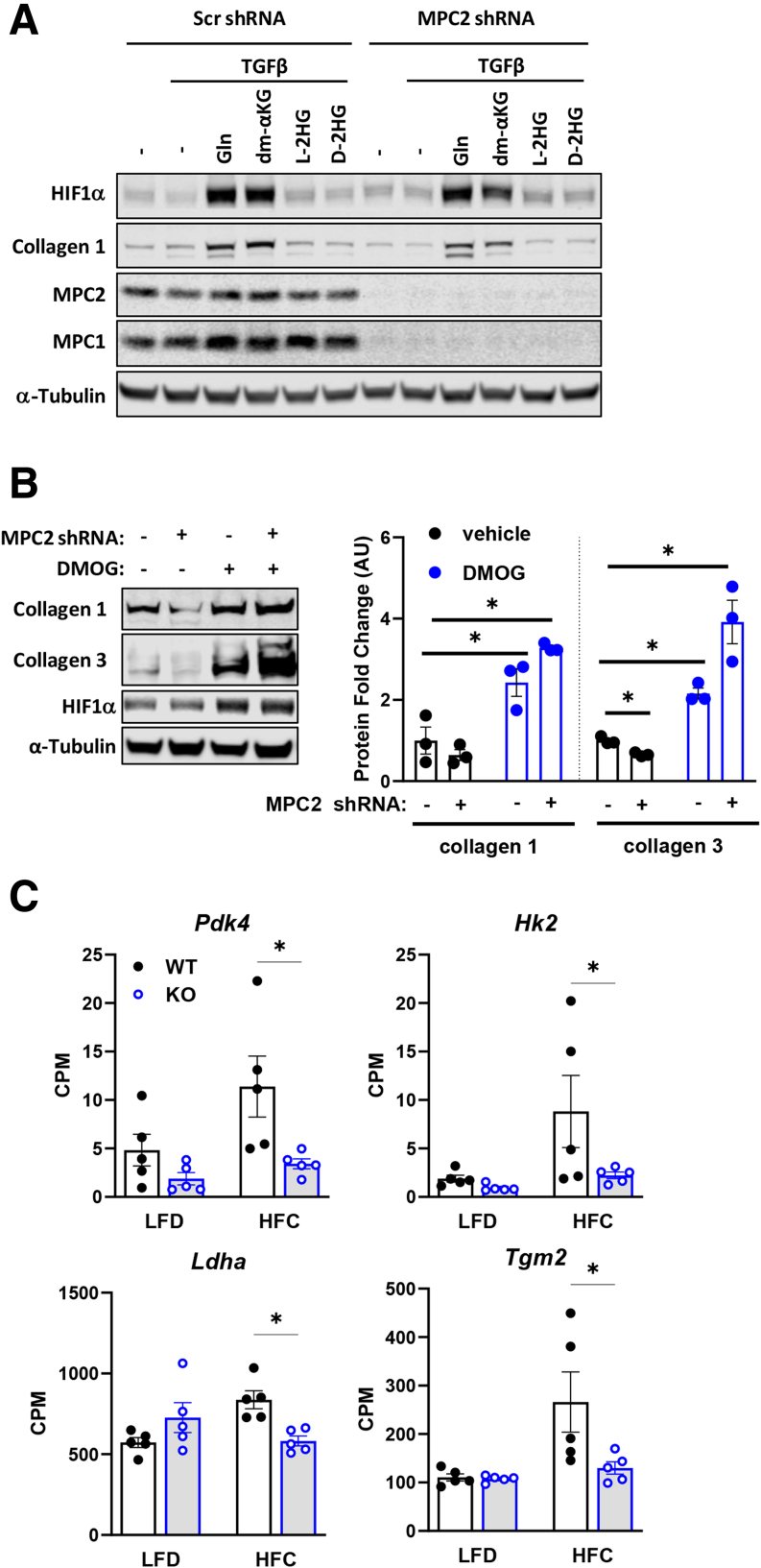
**MPC deficiency leads to impaired HIF1α activation by a metabolic mechanism.** (*A*) The abundance of indicated proteins in LX2 cells treated with or without TGF-β-1 (5 ng/mL), glutamine (Gln, 2 mM), dm-αKG (5 mM), or D- or L-2-hydroxyglutarate (5 mM). (*B*) Protein abundance of collagen 1, collagen 3, and HIF1α in LX2 cells expressing Scr or *MPC2* shRNA treated with TGF-β-1 (5 ng/mL) and with or without DMOG (1 mM), a cell-permeable HIF1α stabilizer. Quantified protein fold change in LX2 cells expressing Scr or shMPC2 treated in the same way. Data expressed as mean ± SEM (n = 3/group) and analyzed by 2-way ANOVA followed by uncorrected Fisher’s LSD test for multiple comparisons; ∗*P* < .05. (*C*) Selected HIF1α target genes from RNAseq data expressed as counts per million (CPM) and represented as mean ± SEM (n = 5/group). ∗*P* < .05 as determined by 2-way ANOVA, followed by Sidak’s test for multiple comparisons. RNA-seq was performed on liver tissue from WT and Lrat-*Mpc2*^-/-^ (KO) mice and placed on either an LFD or HFC diet (n = 5/group).

Because the effect of αKG was somewhat counterintuitive to some known effects of αKG on PHD-mediated hydroxylation and degradation of HIF1α, we tested the effects of dm-αKG supplementation on TCA cycle intermediate abundance and whether other TCA cycle intermediates derived from αKG might stabilize HIF1α. As expected, dm-αKG supplementation increased cellular αKG content in both shMPC2 and control cells (Figure 10*C*). Additionally, dm-αKG supplementation partially corrected the effects of shMPC2 on citrate and aconitate abundance and completely overcame effects on succinate and 2-HG content (Figure 10*C*). However, providing LX2 cells with cell permeable diethyl-succinate, which is known to inhibit PHD,^34^ did not affect HIF1α or collagen protein abundance (Figure 10*D*). Similarly, 2-HG, which can be derived from αKG and stabilize HIF1α,^37^ was without effect on HIF1α or collagen protein abundance (Figure 11*A*). These findings suggest that replenishing TCA cycle intermediates via αKG is sufficient to reverse the effects of MPC2 shRNA on LX2 cell activation. However, we were unable to identify which metabolite might be affecting HIF1α accumulation.

Treating LX2 cells expressing shMPC2 or control shRNA with the prolyl-hydroxylase inhibitor dimethyloxallyl glycine (DMOG) to stabilize HIF1α increased HIF1α, collagen 1, and collagen 3 protein abundance in shMPC2 and scramble control cells (Figure 11*B*). This indicates that HIF1α stabilization is sufficient to overcome the effects of MPC inhibition on LX2 cell activation.

Finally, pathway analyses of liver RNAseq data detected an increase in “hypoxia signaling” in WT mice treated with an HFC diet, and this pathway was downregulated in Lrat-*Mpc2*^-/-^ mice compared with WT mice on the HFC diet (Figure 5*D–E*). Indeed, we detected reduced expression of multiple HIF1α target genes in Lrat-*Mpc2*^-/-^ mice compared with WT mice on the HFC diet (Figure 11*C*), which is consistent with our in vitro mechanistic studies described above. Collectively, these findings suggest that suppression of MPC activity in HSCs attenuates activation by reducing the availability of TCA cycle intermediates to suppress HIF1α signaling.

## Discussion

Activated HSCs undergo complex changes in intermediary metabolism that allow them to proliferate, differentiate into myofibroblasts, and migrate to sites of injury. Prior work has demonstrated rates of glycolysis or glutaminolysis are markedly increased during the activation process, and inhibiting flux through these metabolic pathways can attenuate HSC activation in response to activating stimuli.^13^^,^^17^ However, less is known about other pathways of intermediary metabolism and the therapeutic potential of targeting these pathways to attenuate HSC activation. In the current work, we sought to determine the effects of inhibiting the MPC, which connects anaerobic glycolysis with mitochondrial metabolism by transporting pyruvate, a major end-product of glycolysis, into the mitochondrial matrix where it can enter the TCA cycle as acetyl-CoA or oxaloacetate. Use of MPC inhibitors has been previously shown to reduce markers of HSC activation and fibrosis in a mouse model of MASH,^18^ but the direct effects on HSC activation have been little studied. We found that genetic deletion of MPC2 reduced HSC activation in vitro and in vivo*.* From a mechanistic perspective, we determined that loss of MPC activity reduced the abundance of several TCA cycle intermediates, leading to lower abundance of HIF1α and impaired HSC activation. Importantly, we found that LratCre-*Mpc2*^-/-^ mice were also protected from liver injury and HSC activation in 3 high-fat diet mouse models of MASLD/MASH. Taken together with our previous work showing that MPC inhibitors can reverse established fibrosis in mice,^18^ these studies support the utility of targeting the MPC to attenuate fibrosis in MASH and other chronic liver diseases.

Previous work has suggested that the high rates of glycolysis in HSC are disconnected from mitochondrial pyruvate metabolism.^13^ However, this conclusion was largely based on robust lactate production by HSCs, which can be misleading, rather than assessment of mitochondrial pyruvate flux directly. Indeed, we found that ^13^C-labeled carbons in glucose become highly enriched in TCA cycle intermediates in activated LX2 cells, demonstrating that mitochondrial metabolism of pyruvate is robust. The MPC is required for entry of pyruvate derived from glycolysis into the mitochondria for use in the TCA cycle, and in this study, we show that inhibition of the MPC markedly reduced the pool size (relative abundance) and ^13^C-enrichments of several TCA cycle intermediates. Based on available data, we postulate that inhibition of the MPC limits HSC activation by reducing the ability of cells to use pyruvate as a metabolic substrate, which impacts signaling and biosynthetic processes.

One key metabolite reduced by MPC knockdown was αKG, which has been shown in other types of fibroblasts to play a key role in the activation process.^36^^,^^38^, ^39^, ^40^, ^41^ αKG can be produced from a variety of metabolic processes, including during oxidative metabolism of pyruvate in the TCA cycle, pyruvate transamination to alanine by transaminases, and through the process of glutaminolysis (Figures 4*A* and 5*A*). Prior work has shown that addition of dm-αKG, a cell permeable form of αKG, was able to rescue fibroblast activation with inhibition of glutaminolysis,^17^ and the present findings show that αKG supplementation can also overcome the effects of MPC inhibition or inhibition of glutaminolysis on HSC activation. However, as discussed below, it is not clear whether αKG per se drives activation or if this is a secondary effect of reconstituting TCA cycle intermediates to maintain flux.

The effects of MPC knockdown and diminished TCA cycle activity on HSC activation seem to be mediated, at least in part, via suppression of HIF1α signaling. HIF1α is well-known as a sensor of hypoxic conditions and enhances the expression of enzymes involved in anaerobic glycolysis as an adaptive response to limited oxygen availability.^13^ However, HIF1α is also activated in response to other stimuli and is known to directly regulate the expression of a number of key genes involved in HSC activation.^30^ HIF1α activity can be regulated at multiple levels including at the level of protein stability.^42^, ^43^, ^44^ Under normoxic conditions, HIF1α protein is destabilized by PHDs, which are αKG-dependent dioxygenases that produce succinate as a product of this reaction.^45^^,^^46^ Based on the requirement for αKG in this reaction, it might be expected that low αKG content would reduce PHD activity and therefore stabilization of HIF1α, and prior studies have shown that αKG supplementation reduces HIF1α abundance in some types of fibroblasts.^33^, ^34^, ^35^ However, other work has suggested that HIF1α abundance is increased in human fibroblasts treated with dm-αKG,^36^^,^^47^ which is consistent with the present findings that αKG supplementation could overcome the effects of inhibiting mitochondrial pyruvate import or glutaminolysis. It is likely that downstream metabolites of αKG could be mediating the effects of dm-αKG supplementation. Indeed, metabolomic analyses conducted with dm-αKG supplementation detected a restoration of several TCA cycle and other metabolites, including succinate or 2-HG, which have been shown to suppress PHD activity and promote HIF1α accumulation.^34^^,^^37^ However, although MPC inhibition reduced 2-HG pool sizes, supplementing cells with these intermediates had no effect on HIF1α abundance (Figure 10*D* and Figure 11*A*). It is also possible that other mechanisms or modifications besides HIF1α hydroxylation are mediating this effect, and this should be further dissected in future studies.

Glutaminolysis is critical for the activation process in HSC by providing αKG to the TCA cycle as a metabolic fuel and due to its involvement in proline and other amino acid biosynthesis for collagen production.^14^^,^^48^ Glutamine is the most abundant amino acid in the circulation and an important metabolic substrate. In the present work, we show that αKG supplementation is sufficient to overcome the effects of inhibiting glutaminolysis on collagen protein expression, which highlights the importance of fueling the TCA cycle in this component of the activation process. It is possible that HSCs adapt to decreased pyruvate use as an energy substrate after MPC suppression by diverting the metabolism of this amino acid away from pathways that are required for HSC activation. Indeed, we found that ^13^C-glutamine oxidative metabolism is decreased and reductive glutamine metabolism increased, in response to MPC2 knockdown. This might also be contributing to the observed depletion of TCA cycle intermediates observed with MPC shRNA.

In line with our in vitro findings, we found that mice with stellate cell-specific deletion of *Mpc2* had attenuated liver pathology in response to MASH-inducing diets that was independent of a change in hepatic steatosis. Indeed, Lrat-*Mpc2*^-/-^ mice had reduced blood levels of ALT and AST, reduced hepatic inflammation, and decreased expression of numerous markers of HSC activation. They also exhibited diminished numbers of HSCs based on staining for desmin, but only in the context of the HFC diet. This suggests that the ability to proliferate and activate in response to the HFC diet is impaired. Beyond their involvement in injury responses, HSCs have been shown to regulate liver size, metabolism, and zonation. Previous work demonstrates that HSCs support hepatocyte proliferation by releasing factors such as neurotrophin-3 and R-spondin 3, which regulate hepatocyte proliferation particularly in zone 2 of the liver.^49^^,^^50^ Specific depletion of HSCs reduced liver size and altered various metabolic transcriptional profiles. Along these lines, we observed reductions in liver-to-body weight ratios in Lrat-*Mpc2*^-/-^ mice on MASH-inducing diets, and our bulk RNAseq data revealed that *Mpc2* deletion in HSC altered fatty acid and bile acid metabolism.

Further analysis of bulk RNAseq data revealed a striking decrease in gene sets associated with both innate and adaptive immune responses. This is a key finding because immune cell infiltration is one of the early signs of MASLD development.^51^ Examination of scRNAseq data revealed that the changes in immune responses were most likely driven by alterations in myeloid cell clusters, including subsets of monocytes, macrophages, and dendritic cells. Accordingly, we found decreased percentages of LAMs and C-LAMs, which are comparable to macrophages from obese adipose tissue^52^ and form hepatic crown-like structures in areas that are abundant in stellate cells.^27^^,^^53^ In contrast to our bulk RNAseq data, we found that many cell types had increased interferon responses and JAK-STAT signaling pathways. This could be due to the differences in diets used for bulk and scRNAseq (HFC and CDAA, respectively). Nevertheless, both diet regimens revealed reduced markers of fibrosis and HSC activation in the Lrat-*Mpc2*^*-/-*^ mice. Additionally, our single-cell analysis only included non-parenchymal cells, whereas our bulk RNAseq data was from whole liver. Previous work has demonstrated that hepatocyte interferon expression is increased in response to HFD.^54^ Because hepatocytes make up the majority of cells within the liver, it is possible that interferon expression in hepatocytes, which were absent in our single-cell data, could be driving the results of our bulk RNAseq analysis. Unfortunately, we found only a few HSCs in our scRNAseq data, which could be due to multiple factors. In comparison to other hepatic cell types, HSCs are not highly abundant and represent only 1% to 5% of cells in the liver.^55^ We also elected to use mice that were on a CDAA diet, which induces fibrosis in mice in about 8 to 12 weeks. It is possible that our collagenase digestion was not stringent enough to allow for separation of the HSC from extracellular components in more fibrotic livers. However, we did detect reductions in the expression of enzymes involved in the TCA cycle, oxidative phosphorylation, and extracellular matrix organization, which align with our in vitro data.

In summary, the current work provides evidence that inhibition of the MPC ameliorates HSC activation by altering intermediary metabolism to affect HIF1α signaling. Using Cre-LoxP mediated recombination, we generated novel stellate cell-specific *Mpc2* knockout mice, which were protected from hepatic injury and HSC activation in 2 separate dietary models. Analysis of hepatic RNAseq data demonstrated that HSC-specific *Mpc2* deletion resulted in decreased expression of pathways associated with immune cell activation and extracellular matrix synthesis. Overall, these data highlight an alternative mechanism by which MPC inhibitors could be a novel therapeutic option for people afflicted with MASH.

## Materials and Methods

### Animal Studies

All animal experiments were approved by the Institutional Animal Care and Use Committee of Washington University in St. Louis. Male or male and female mice in the C57BL/6J background were used in our animal experiments as indicated. To generate HSC-specific deletion of *Mpc2*, we crossed *Mpc2*^fl/fl^ mice^21^ with lecithin retinol acyltransferase-Cre (Lrat-Cre).^6^ Littermates that did not express *Cre* were used as controls in all studies.

For HFD studies shown in Figure 2, 8-week-old male mice were fed with an LFD (Research Diets D09100304) or an HFC diet (42% kcal fat, 20% fructose, 2% cholesterol; Research Diets D09100310) for a period of 12 weeks. Four weeks after the initiation of experimental diets (ie, 12 weeks of age), mice received a single intraperitoneal injection of carbon tetrachloride dissolved in corn oil (0.5 μL carbon tetrachloride per gram body weight). Finally, after 12 weeks on diet, animals were fasted for 5 hours and euthanized by using CO_2_ asphyxiation, and tissue samples were collected for further analyses. Blood samples were collected through the inferior vena cava into EDTA-containing tubes.

A cohort of 10- to 11-week old male and female HSC-specific *Mpc2* knockout mice and WT littermates were fed either a CDAA diet (45 kcal% fat with 0.1% methionine and no added choline; A06071309; Research Diet Inc.) or a chow control diet for 10 weeks. On week 11, animals were fasted for 5 hours, then euthanized using CO_2_ asphyxiation, and tissue samples collected for further analyses. Blood samples were collected into EDTA-containing tubes, and plasma was stored at −80 °C for further analyses.

### HSC Isolation

Primary HSCs were isolated from mice as previously described.^18^ Briefly, following pronase and collagenase perfusion of the liver, hepatocytes were removed from the cell suspension using a short centrifugation (50 × *g* for 2 minutes at 4 °C). Then HSCs were purified using a density gradient centrifugation using Optiprep (Sigma), and cells were seeded on to standard tissue culture dishes in Dulbecco’s Modified Eagle Medium (DMEM) (Gibco) containing 10% fetal bovine serum (FBS), and 1× peniicillin/streptomycin. After 24 hours, a subset of cells was harvested for use as a quiescent HSC control group. Other cells remained in culture for 7 days before RNA was isolated as described below. Media was replenished every other day during the 7-day period.

### Cell Death

Cell death analysis was performed on primary hepatic stellate cells isolated from Lrat-*Mpc2*^*-/-*^ and WT littermate mice. Cells were seeded in a black, clear-bottom 96-well plate and incubated overnight at 37 °C. The following day, cells were washed with pre-warmed phenol red-free DMEM and incubated for 1 hour with 500 nM SYTOX Green nucleic acid stain (S7020; Invitrogen) in the same medium. Fluorescent images were captured using an EVOS M5000 microscope (Invitrogen), and dead cells were quantified with ImageJ (Fiji) software (National Institutes of Health).

### LX2 Experiments

The LX2 human stellate cell line (LX2 cells; Millipore Sigma, SCC064) was maintained in DMEM (Gibco, 11965-084) supplemented with 2% FBS, sodium pyruvate (1 mM), and penicillin-streptomycin (100 U/mL). Unless otherwise indicated, LX2 cells were cultured overnight with indicated treatments in Gln-free DMEM (Gibco, 11960-051) containing 10% FBS, 1 mM sodium pyruvate, and 100 U/mL penicillin-streptomycin. Reagents and inhibitors used in these studies included: TGF-β-1 (PeproTech; AF-100-21C), dm-αKG (Sigma-Aldrich; 349631), diethyl-succinate (Sigma-Aldrich; 112402), D-α-hydroxyglutaric acid (Millipore-Sigma; 16859), L-α-hydroxyglutaric acid (Millipore-Sigma; 90790), BPTES (Cayman; 19284), CB-839 (MedChemExpress; HY-12248), and DMOG (MedChemExpress; HY-15893).

For Seahorse assays, LX2 cells were seeded at 4 × 10^4^ cells/well in an Agilent Seahorse XF24 Microplate in DMEM with 2% FBS and incubated overnight at 37 °C with 5% CO_2_. On the next day, the medium was replaced with 500 ul fresh XF Base Medium (supplemented with 10 mM glucose, 1 mM pyruvate, 2 mM glutamine, pH 7.4), and cells were treated accordingly and incubated overnight at 37 °C in a non-CO_2_ incubator. On the assay day, oxygen consumption rate (OCR) was measured using an Agilent Seahorse XFe24 Bioanalyzer with sequential injections of 1.5 μM oligomycin, 1.0 μM FCCP, and 1.0 μM rotenone/antimycin A to assess mitochondrial parameters. Data were normalized to cell number, analyzed with Wave software (Agilent), and expressed as mean ± standard error of the mean (SEM).

### Lentivirus Transfection

Lentiviral vectors expressing shRNAs against human MPC2, and lentiviral control non-targeting vector expressing scrambled shRNA were developed by FenicsBIO (MD). The shRNA sequences used in this study were: Lentiviral human MPC2 shRNA-1 (CMV) (Cat. HSH-518021-1), 5′-AAGATACTCACTTGTAATTATT-3′, Lentiviral human MPC2 shRNA-2 (CMV) (Cat. HSH-518021-2), 5′-GCCAGACCTGCAGAAAAACTTA-3′, and lentiviral control shRNA (CMV) (Cat. SH-CMV-C01). Following the infection of LX2 cells with lentiviral MPC2-targeting and non-targeting control vectors, puromycin (Sigma-Aldrich, P4512) was administered for stable selection of the cells expressing the shRNAs.

### Immunoblotting

RIPA lysis buffer (Cell Signaling Technology; 9806) with protease/phosphatase inhibitor cocktail (Cell Signaling Technology; 5872) was used to extract protein from liver or cultured cells as previously described.^56^ For in vitro samples, cells were washed 1× with ice-cold phosphate-buffered saline (PBS), and lysis buffer was added in each well. Cells were scraped, briefly sonicated, and centrifuged for 10 minutes at 14,000 RPM at 4 °C. For liver samples, approximately 30 mg of liver was homogenized using a TissueLyser and stainless steel beads. Protein was quantified using BCA assay (Thermo Scientific; 23227). Then, samples were mixed with 4× sample buffer (Invitrogen; NP0007) with β-mercaptoethanol (BioRad; 1610710). Proteins were heat denatured, loaded on NuPAGE precast 4% to 12% Bis-Tris gels (Invitrogen; NP0322BOX; NP0329BOX; NP04122BOX), and run with MOPS (Invitrogen; NP0001) or MES (Invitrogen; NP0002) buffer and transferred to PVDF membrane (Sigma-Aldrich; IPFL00010). Antibodies used included: collagen 1 (Cell Signaling Technology; 72026), collagen 3 (ProteinTech; 22734-1-AP), HIF1-α (Cell Signaling Technology; 36169), MPC1 (Cell Signaling Technology; D2L9I), MPC2 (Cell Signaling Technology; D4I7G), smooth muscle actin (SMA; Sigma-Aldrich; CBL171), α-tubulin (Sigma-Aldrich; T5168), β-ACTIN (Cell Signaling Technology; 3700), and COXIV (Cell Signaling Technology; 11967). To obtain Western blot images, a Licor system was used with Image StudioLite software.

### Isotope-tracing Experiments

LX2 cells expressing shMPC2 or scrambled shRNA control were incubated overnight in DMEM containing 25 mM U-[^13^C]-glucose (Cambridge Isotope Laboratories, CLM-1396-PK) or 25 mM U-[^13^C]-glutamine (Cambridge Isotope Laboratories, CLM-1822-H-PK), 10% FBS, 2 mM glutamine, 1 mM sodium pyruvate, and 1% penicillin/streptomycin and treated with 5 ng/mL TGF-β-1. Samples were collected and prepared for intracellular metabolite measurement as previously described.^56^ Cells were washed one time with pre-warmed PBS and one time with pre-warmed HPLC-grade water. Then, quenched cells with ice-cold HPLC-grade methanol were scraped and collected into Eppendorf tubes followed by SpeedVac drying for 2 to 3 hours. After being reconstituted in 1 mL of ice-cold methanol:acetonitrile:water (2:2:1), all samples were vortexed, frozen in liquid nitrogen, and sonicated for 10 minutes at 25 °C for 3 consecutive cycles. The samples were then centrifuged at 14,000 × *g* at 4 °C after being kept at −20 °C for at least 1 hour. Supernatants were transferred to fresh tubes and SpeedVac dried for 2 to 5 hours. The protein content of cell pellets was quantified by using a BCA kit from ThermoFisher to standardize across samples. Following the drying of the supernatant, 1 mL of water:acetonitrile (1:2) was added per 2.5 mg of cell protein, as measured in pellets recovered after extraction. Samples were subjected to 2 cycles of vortexing and 10 minutes of sonication at 25 °C. Finally, samples were centrifuged at 14,000 × *g* and 4 °C for 10 minutes, the supernatant was transferred to liquid chromatography (LC) vials and kept at −80 °C until LC/mass spectrometry (MS) analysis.

For studies shown in Figure 6*C*, LX2 cells expressing shMPC2 or scrambled shRNA control were incubated overnight in a high-glucose-glutamine-free DMEM containing 10% FBS, 1 mM sodium pyruvate, and 1% penicillin/streptomycin and treated with/without 5 ng/mL TGFβ-1 and 5 mM dm-αKG (Sigma-Aldrich, 349631). Samples were collected and prepared for intracellular metabolite measurement as described just above.

Ultra-high-performance LC (UHPLC)/MS was performed as previously described^57^ with a ThermoScientific Vanquish Flex UHPLC system interfaced with a ThermoScientific Orbitrap ID-X Tribrid Mass Spectrometer. Hydrophilic interaction liquid chromatography (HILIC) separation was accomplished by using a HILICON iHILIC-(P) Classic column (Tvistevagen) with the following specifications: 100 × 2.1 mm, 5 μm. Mobile-phase solvents were composed of A = 20 mM ammonium bicarbonate, 0.1% ammonium hydroxide and 2.5 μM medronic acid in water:acetonitrile (95:5) and B = in acetonitrile:water (95:5). The column compartment was maintained at 45 °C for all experiments. The following linear gradient was applied at a flow rate of 250 μL min^−1^: 0 to 1 minute: 90% B, 1 to 12 minutes: 90% to 35% B, 12 to 12.5 minutes: 35% to 25% B, 12.5 to 14.5 minutes: 25% B. The column was re-equilibrated with 20-column volumes of 90% B. The injection volume was 2 μL for all experiments. Data were collected with the following settings: spray voltage, −3.0 kV; sheath gas, 35; auxiliary gas, 10; sweep gas, 1; ion transfer tube temperature, 250 °C; vaporizer temperature, 300 °C; mass range, 67 to 1500 Da, resolution, 120,000 (MS1), 30,000 (MS/MS); maximum injection time, 100 ms; isolation window, 1.6 Da. LC/MS data were processed and analyzed with the open-source Skyline software.^58^

### RNA Isolation and Quantitative Real-time Polymerase Chain Reaction

RNA was isolated as previously described.^59^ For in vitro studies, RNA was harvested from tissue culture dishes using TRIzol Reagent (Ambion) and Purelink RNA Mini Kit (Invitrogen). For liver tissue, ∼30 mg of frozen liver tissue was homogenized in TRIzol Reagent (Ambion) using the TissueLyser II (Qiagen) followed by isolation using the Purelink RNA Mini Kit (Invitrogen). RNA was reverse transcribed into complimentary DNA (cDNA) using High-Capacity reverse transcriptase (Life Technologies). Quantitative real-time polymerase chain reaction (qRT-PCR) was performed using Power SYBR Green (Thermo Fisher Scientific) and an optical 384-Well Reaction Plate (Applied Biosystems) using ViiA 7 Real-Time PCR System (Applied Biosystems). Relative gene expression was determined by the ΔΔCt method *36b4* as a reference gene. The primer sequences applied for gene expression are available in Supplementary Table 2.

### RNA-seq

RNA was isolated from liver tissue as described above, and bulk RNA-seq was performed at the Genomic Technologies and Access Center at the McDonald Genomic Institute of Washington University School of Medicine in St. Louis. An Agilent Bioanalyzer was used to assess RNA integrity, and ribosomal RNA depletion was performed with RiboErase (HMR). Samples were prepared according to library kit manufacturer’s protocol, then indexed, pooled, and sequenced on a NovaSeq S4 2x150, targeting ∼30 million reads per sample. Detailed methods of RNA-seq analysis are previously reported.^59^

### ScRNA-seq

Livers were enzymatically digested as described previously.^53^^,^^60^ Briefly, following euthanasia via carbon dioxide, PBS was perfused through the portal vein for ∼3 minutes. Liver tissue was finely minced and digested at 37 °C for 30 minutes with rotation in a collagenase buffer (DMEM, collagenase A [0.75 mg/mL], and DNaseI [50 ug/mL]). Digests were placed through a 70-μm cell strainer along with FBS containing DMEM, then centrifuged at 50 × g for 3 minutes at 4 °C to remove hepatocytes. The supernatant (non-parenchymal cells) was pelleted at 163 × g for 7 minutes at 4 °C, then ACK lysis buffer was used to lyse red blood cells, washed with PBS, and re-pelleted by centrifugation. The cell pellet was incubated with Zombie Aqua solution for live/dead staining, then washed and resuspended in FACS buffer (PBS, 0.5% bovine serum albumin [BSA], 2 mM EDTA). Single live cells were collected using the Aria II-1 cell sorter, resuspended in PBS with 0.04% BSA, and submitted to the Genome Technology Access Center (GTAC) at Washington University, which used the Chromium GEM-X 3′ kit v4 (target 20,000 cells), and Chromium instrument (10X Genomics) to tag polyadenylated mRNA from individual cells with a unique 16-bp 10X barcode and a 10-bp Unique Molecular Identifier (UMI). Following library quality control assessment, Illumina sequencing (Nova-seq) was performed at the McDonnel Genome Institute sequencing center, where samples were sequenced at a depth of 50,000 reads/cell.

The CellRanger filtered h5 expression matrices for each sample was loaded into Seurat v5 and filtered cells for numbers of genes with less than 25th percentile for the lower limit, 1.5 times the interquartile range (IQR) for the upper limit of expressed genes for each cell compared to all cells, and greater than 10 percent mtRNA. Ambient RNA was then removed with SoupX, doublets were removed with scDblFinder, and filtered adjusted counts for each sample was then normalized with SCTransform v2. We then clustered the cells with Seurat’s Louvain algorithm with a 0.8 resolution. Each sample’s Seurat object was merged into a new layered multi-sample Seurat object that was then integrated with Seurat’s reciprocal principal component analysis (PCA) method and then split by experimental condition. Clusters were annotated based on previously established cell markers.^27^^,^^53^

DEGs were determined using the Seurat (v5.1.0) *FindMarkers* function.^61^ Hallmark pathway analysis was performed using the *compare_pathways* function in the SCPA (v1.6.2) package^24^ using default parameters. ComplexHeatmap (v2.22.0) was used for SCPA visualization. Gene set activity for pathways of interest, identified from the SCPA analysis, were assessed at the single-cell level using the *AddModuleScore*^62^ function in Seurat (v5.1.0). Statistical significance was determined using the Wilcoxon rank-sum test.

### Body Composition

Total body composition of fat and lean mass was determined by EchoMRI-100H (EchoMRI LLC).

### Plasma Analyses

Plasma levels of ALT (Teco Diagnostics; A524150) and AST (Teco Diagnostics; A7561450) were determined using kinetic absorbance assays as described previously.^18^ Analysis of plasma triglycerides (Thermo Fisher Scientific; TR22421), total cholesterol (Thermo Fisher Scientific; EEA026), and non-esterified fatty acids (FUJIFILM Wako; NEFA-HR(2)) were determined using colorimetric based assay as previously reported.^63^^,^^64^

### Hepatic Triglyceride and Nonesterified Fatty Acid Analyses

Frozen liver samples were weighed (50-100 mg) and homogenized in ice-cold PBS (Gibco, 14190-051). Then, hepatic lipids were solubilized in 1% sodium deoxycholate and using the commercially available kits, hepatic triglyceride (Thermo Scientific, TR22421) and nonesterified fatty acids (NEFA) (FUJIFILM Wako; NEFA-HR (2)) were quantified according to the manufacturer’s directions.

### Histologic Analyses

Liver tissue was fixed in 10% neutral buffered formalin for 24 hours then paraffin embedded, then sections were cut and stained with H&E. H&E sections were assessed for steatosis score, lobular inflammation, and NAFLD total score, by a board-certified pathologist (M.H.) who was blinded from treatment groups, as previously reported.^18^^,^^64^

### Immunofluorescence and Quantification

Livers were fixed in 10% formalin for 24 hours at 4 °C, then washed and incubated in 30% sucrose for another 24 hours at 4 °C. The specimens were then embedded in OCT compound, cut into 8-μm thick sections by a cryostat (Leica Biosystems), and stored at −80 °C. For staining, sections were air-dried for 12 minutes, rehydrated in PBS for 5 minutes, and then blocked with freshly prepared blocking buffer (PBS +1% BSA w/v +0.3% Triton X-100 v/v) for 1 hour at room temperature. After blocking, 50 μL of primary antibody cocktail prepared in blocking buffer was deposited on the section for overnight incubation at 4 °C in a humidified chamber. The antibody solution was then aspirated, and the sections were washed with PBS, 3 times, 5 minutes each. The secondary antibody cocktail was then similarly deposited on the section and incubated for 1 hour at room temperature in the dark. Sections were then washed 3 times with PBS, 5 minutes each, and nuclei were stained with fresh Hoechst dye (1:25,000, in PBS) for 5 minutes in the dark at room temperature. The sections were then mounted with prolong gold antifade mounting reagent and a #1 coverslip. Antibodies used were: F4/80 (eBioscience 13-4801-85; 1:200), desmin (Abcam Ab15200; 1:100), anti-Rat IgG AF488 (Invitrogen A21208; 1:500), and anti-Rabbit IgG (Jackson ImmunoResearch 711-585-152; 1:200).

Confocal images were acquired using an LSM 900 laser scanning confocal microscope (ZEISS) with a 20× 0.8 N.A. objective at ambient temperature. All the images were acquired in the same period using the same laser intensity and gain. The brightness of individual channels was optimized uniformly in Fiji (ImageJ, National Institutes of Health) by adjusting minimum and maximum displayed values. Quantification on the desmin-positive area was performed blindly using manual thresholding. Five randomly chosen fields (excluding large vessels) per sample were acquired for quantification.

### Statistical Analyses

GraphPad Prism Software, version 9.5.0 for windows, was used to generate figures. All data and are presented as the mean ± SEM. Statistical significance was calculated using an unpaired Student *t*-test, 2-way analysis of variance (ANOVA), or 1-way ANOVA with Tukey’s multiple comparisons test, with a statistically significant difference defined as *P* < .05.
