# Supplementary material for: A Critical Role for the Mitochondrial Pyruvate Carrier in Hepatic Stellate Cell Activation
Source: Cell Mol Gastroenterol Hepatol. 2025 Apr 14;19(8):101517. doi: 10.1016/j.jcmgh.2025.101517 (PMC12166444; doi:10.1016/j.jcmgh.2025.101517)
Supplement: Supplementary Table S1-S3 [file mmc1.docx]

**Table S1. Primer sequences applied for gene expression**

| **Human Primers** |  | **Forward 5'-3'** |  | **Reverse 5'-3'** |
| --- | --- | --- | --- | --- |
| *Col1a1* |  | gag ggc caa gac gaa gac atc |  | cag atc acg tca tcg cac aac |
| *Col1a2* |  | ggc cct caa ggt ttc caa gg |  | cac cct gtg gtc caa caa ctc |
| *Col3a1* |  | gga gct ggc tac ttc tcg c |  | ggg aac atc ctc ctt caa cag |
| *Fn1* |  | gat aaa tca aca gtg gga gc |  | ccc aga tca tgg agt ctt ta |
| *Hif1α* |  | agc cga gga aga act atg aac |  | att tga tgg gtg agg aat ggg |
| *Gpt2* |  | cag gcg cgc gag cta ac |  | gcg cgc ttg cgg aga g |
| *Mpc1* |  | gtg cgg aaa gcg gcg gac ta |  | ggc agc aat ggg aag acc cca |
| *Mpc2* |  | tac cac cgg ctc ctc gat aaa |  | tat cag cca atc cag cac aca |
| *36B4* |  | gtg atg tgc agc tga tca aga ct |  | gat gac cag ccc aaa gga ga |
|  |  |  |  |  |
| Mouse Primers |  | **Forward 5'-3'** |  | **Reverse 5'-3'** |
| *Acta2* |  | gtc cca gac atc agg gag taa |  | tcg gat act tca gcg tca gga |
| *Col1a1* |  | gct cct ctt agg ggc cac t |  | cca cgt ctc acc att ggg g |
| *Col1a2* |  | gta act tcg tgc cta gca aca |  | cct ttg tca gaa tac tga gca gc |
| *Col3a1* |  | ctg taa cat gga aac tgg gga aa |  | cca tag ctg aac tga aaa cca cc |
| *Timp1* |  | gca act cgg acc tgg tca taa |  | cgg ccc gtg atg aga aac t |
| *SPP1* |  | atc tca cca ttc gga tga gtc t |  | tgt agg gac gat tgg agt gaa a |
| *Mpc2* |  | ccg ccg cga tgg cag ctg |  | gct agt cca gca cac acc aat cc |
| *Lgals3* |  | gtt gcg gtc aac gat gct c |  | tga tcc cca gtt ggc tga ttt |
| *Serpine1* |  | gcc agg gtt gca cta aac at |  | gcc tcc tca tcc tgc cta a |
| *36B4* |  | gca gac aac gtg ggc tcc aag cag at |  | ggt cct cct tgg tga aca cga agc cc |

**Table S2.** Metabolite pool size fold changes of TGFβ-induced stable LX2 cells expressing shMPC2 compared to scramble control. Cells were treated with ^13^C-glucose or ^13^C-glutamine.

| Compound |  | MPC2 Fold changes |  | S.E.M. |  | *p*-value |
| --- | --- | --- | --- | --- | --- | --- |
| 2/3-PG |  | 1.05 |  | 0.08 |  | 0.644 |
| 2-hydroxyglutaric acid |  | 0.78 |  | 0.06 |  | 0.017 |
| 2KIVA |  | 0.79 |  | 0.06 |  | 0.018 |
| 3-Indoleacrylic acid |  | 1.18 |  | 0.08 |  | 0.059 |
| 4-Hydroxyprolylglutamine |  | 0.43 |  | 0.04 |  | <0.001 |
| 6-Phosphogluconic acid |  | 0.81 |  | 0.09 |  | 0.101 |
| Acetylcarnitine |  | 0.36 |  | 0.03 |  | <0.001 |
| Acetyl-CoA |  | 0.46 |  | 0.08 |  | 0.017 |
| Aconitate |  | 0.31 |  | 0.04 |  | <0.001 |
| Adenine |  | 1.21 |  | 0.12 |  | 0.160 |
| Adenosine |  | 1.54 |  | 0.19 |  | 0.018 |
| ADP |  | 1.09 |  | 0.12 |  | 0.513 |
| αKG |  | 0.57 |  | 0.08 |  | 0.002 |
| Alanine |  | 0.96 |  | 0.05 |  | 0.557 |
| AMP |  | 1.05 |  | 0.16 |  | 0.802 |
| Arginine |  | 1.04 |  | 0.04 |  | 0.521 |
| Asparagine |  | 0.83 |  | 0.08 |  | 0.080 |
| Aspartate |  | 1.18 |  | 0.05 |  | 0.042 |
| ATP |  | 1.02 |  | 0.04 |  | 0.758 |
| Carnitine |  | 1.16 |  | 0.10 |  | 0.142 |
| CDP |  | 0.95 |  | 0.11 |  | 0.695 |
| Citrate |  | 0.58 |  | 0.05 |  | <0.001 |
| Creatine |  | 1.06 |  | 0.04 |  | 0.220 |
| CTP |  | 1.01 |  | 0.05 |  | 0.889 |
| CDP-Choline |  | 0.86 |  | 0.04 |  | 0.126 |
| CMP |  | 1.13 |  | 0.06 |  | 0.415 |
| Cysteine-glutathione disulfide |  | 0.94 |  | 0.24 |  | 0.834 |
| Cytidine |  | 0.69 |  | 0.09 |  | 0.148 |
| DHAP |  | 1.51 |  | 0.17 |  | 0.019 |
| FAD |  | 0.98 |  | 0.04 |  | 0.745 |
| Fructose-bisphosphate |  | 1.66 |  | 0.10 |  | <0.001 |
| Fumarate |  | 0.90 |  | 0.02 |  | 0.064 |
| GlcNAc-Phosphate |  | 1.23 |  | 0.12 |  | 0.310 |
| Glucose |  | 1.05 |  | 0.28 |  | 0.877 |
| Glucose-6-phosphate |  | 0.85 |  | 0.05 |  | 0.058 |
| Glu-gln |  | 1.14 |  | 0.09 |  | 0.379 |
| Glutamate |  | 0.87 |  | 0.02 |  | 0.001 |
| Glutamine |  | 0.96 |  | 0.01 |  | 0.315 |
| Glutathione, oxidized |  | 1.34 |  | 0.24 |  | 0.233 |
| Glutathione, reduced |  | 1.20 |  | 0.19 |  | 0.354 |
| Glycerol |  | 0.90 |  | 0.09 |  | 0.411 |
| Glycerol 3-phosphate |  | 2.15 |  | 0.11 |  | <0.001 |
| Glycine |  | 1.23 |  | 0.07 |  | 0.048 |
| Guanosine |  | 1.44 |  | 0.12 |  | 0.062 |
| Histidine |  | 1.26 |  | 0.06 |  | 0.011 |
| Lactate |  | 0.98 |  | 0.07 |  | 0.874 |
| Leu/Ile |  | 1.32 |  | 0.08 |  | 0.002 |
| Lysine |  | 1.07 |  | 0.05 |  | 0.272 |
| Malate |  | 0.95 |  | 0.02 |  | 0.450 |
| Methionine |  | 1.14 |  | 0.05 |  | 0.019 |
| Methylmalonate |  | 0.59 |  | 0.15 |  | 0.188 |
| N,N-Dimethylarginine |  | 1.03 |  | 0.08 |  | 0.688 |
| NAD+ |  | 1.00 |  | 0.05 |  | 0.970 |
| NADH |  | 0.93 |  | 0.15 |  | 0.732 |
| NADP+ |  | 1.06 |  | 0.06 |  | 0.337 |
| NADPH |  | 0.76 |  | 0.23 |  | 0.350 |
| Nicotinamide Mononucleotide |  | 0.93 |  | 0.07 |  | 0.472 |
| Pentose-5-phosphate |  | 1.32 |  | 0.12 |  | 0.028 |
| Phenylalanine |  | 1.20 |  | 0.07 |  | 0.019 |
| Phosphocreatine |  | 1.03 |  | 0.03 |  | 0.339 |
| Phosphoenolpyruvate |  | 1.47 |  | 0.26 |  | 0.116 |
| Proline |  | 1.05 |  | 0.07 |  | 0.486 |
| pRpp |  | 0.79 |  | 0.08 |  | 0.083 |
| Pyruvate |  | 1.04 |  | 0.21 |  | 0.864 |
| S-Adenosylmethionine |  | 1.37 |  | 0.07 |  | 0.002 |
| Sedoheptulose 7-phosphate |  | 1.19 |  | 0.11 |  | 0.122 |
| Serine |  | 1.06 |  | 0.03 |  | 0.158 |
| Succinate |  | 0.69 |  | 0.11 |  | 0.039 |
| Threonine |  | 1.05 |  | 0.04 |  | 0.320 |
| Tryptophan |  | 1.18 |  | 0.09 |  | 0.068 |
| Tyrosine |  | 1.16 |  | 0.06 |  | 0.024 |
| UDP |  | 1.01 |  | 0.09 |  | 0.912 |
| UDP-glucose / UDP-galatose |  | 0.89 |  | 0.03 |  | 0.013 |
| UDP-N-acetylglucosamine |  | 0.74 |  | 0.07 |  | 0.007 |
| Uracil |  | 1.21 |  | 0.07 |  | 0.155 |
| Uridine |  | 1.03 |  | 0.11 |  | 0.828 |
| Uridine 5'-diphosphoglucuronic acid |  | 0.73 |  | 0.06 |  | 0.004 |
| UTP |  | 1.03 |  | 0.06 |  | 0.691 |
| Valine |  | 1.27 |  | 0.08 |  | 0.007 |

**Table S3.** Metabolite pool size fold changes (FC) of TGFβ-induced stable LX2 cells expressing shMPC2 compared to scramble control. Cells were treated with or without dm-αKG.

| Compound | shScr | shMPC2 | shScr + αKG | shMPC2 + αKG | SEM |
| --- | --- | --- | --- | --- | --- |
| Glucose | 1.00 | 0.58 | 1.80^*^ | 1.20^¥€^ | 0.25 |
| Glucose-6-phosphate | 1.00 | 1.14 | 1.08 | 0.93 | 0.05 |
| Fructose-bisphosphate | 1.00 | 1.30 | 0.86 | 0.94 | 0.10 |
| DHAP | 1.00 | 1.21 | 0.40^*^ | 1.36^¥^ | 0.21 |
| 2-PG | 1.00 | 1.01 | 1.35 | 1.08 | 0.08 |
| 3-PG | 1.00 | 0.92 | 1.43 | 1.01 | 0.11 |
| Phosphoenolpyruvate | 1.00 | 0.81 | 2.40^*^ | 1.12^¥^ | 0.36 |
| Pyruvate | 1.00 | 0.97 | 1.53 | 1.02 | 0.13 |
| Lactate | 1.00 | 1.02 | 1.06 | 0.89 | 0.04 |
| Mannitol/Sorbitol | 1.00 | 0.82 | 1.46 | 1.11 | 0.13 |
| Myoinositol | 1.00 | 1.03 | 0.76 | 0.51 | 0.12 |
| Glucose-1-phosphate/Fructose-6-phosphate | 1.00 | 0.94 | 1.00 | 0.93 | 0.02 |
| Mannose-6-phosphate | 1.00 | 0.99 | 1.19 | 0.96 | 0.05 |
| Ribose-5-phosphate | 1.00 | 1.10 | 0.98 | 0.72 | 0.08 |
| Xylulose-5-phosphate | 1.00 | 1.75^*^ | 0.65 | 1.45^¥^ | 0.24 |
| Ribulose-5-phosphate | 1.00 | 1.21 | 1.22 | 1.34 | 0.07 |
| 6-Phosphogluconic acid | 1.00 | 1.01 | 3.07^*^ | 2.23^*¥€^ | 0.51 |
| Sedoheptulose 7-phosphate | 1.00 | 1.17 | 2.53^*^ | 1.91^*¥€^ | 0.35 |
| Citrate | 1.00 | 0.73 | 1.83^*^ | 1.22^¥^ | 0.23 |
| Aconitate | 1.00 | 0.58 | 1.65^*^ | 1.21^€^ | 0.22 |
| αKG | 1.00 | 0.71 | 4.08^*^ | 4.26^*€^ | 0.96 |
| Succinate | 1.00 | 0.79 | 4.40^*^ | 3.43^*¥€^ | 0.90 |
| Fumarate | 1.00 | 0.88 | 1.03 | 1.01 | 0.03 |
| Malate | 1.00 | 0.61 | 2.73^*^ | 1.95^*¥€^ | 0.48 |
| Glutamate | 1.00 | 0.66 | 2.09^*^ | 1.38^¥€^ | 0.31 |
| Glutamine | 1.00 | 0.72 | 5.66^*^ | 4.05^*¥€^ | 1.20 |
| Aspartate | 1.00 | 0.52 | 1.88^*^ | 1.52^€^ | 0.30 |
| Alanine | 1.00 | 0.80 | 0.95 | 0.83 | 0.05 |
| β-Alanine | 1.00 | 1.15 | 1.23 | 1.13 | 0.05 |
| Glycine | 1.00 | 1.20 | 0.80 | 0.84 | 0.09 |
| Valine | 1.00 | 1.32 | 0.68 | 0.86 | 0.13 |
| Leucine | 1.00 | 1.38 | 0.60 | 0.70^€^ | 0.17 |
| Isoleucine | 1.00 | 1.21 | 0.47 | 0.97 | 0.16 |
| Asparagine | 1.00 | 0.95 | 1.27 | 1.05 | 0.07 |
| Proline | 1.00 | 1.19 | 0.88 | 0.79 | 0.09 |
| Histidine | 1.00 | 1.11 | 0.55 | 0.67 | 0.13 |
| Methionine | 1.00 | 1.19 | 0.67 | 0.80 | 0.11 |
| Lysine | 1.00 | 1.55 | 0.86 | 0.78^€^ | 0.17 |
| Arginine | 1.00 | 1.22 | 0.87 | 0.83 | 0.09 |
| Phenylalanine | 1.00 | 1.24 | 0.66 | 0.68^€^ | 0.14 |
| Serine | 1.00 | 0.98 | 0.94 | 0.77 | 0.05 |
| Threonine | 1.00 | 1.05 | 0.88 | 0.82 | 0.05 |
| Tyrosine | 1.00 | 1.26 | 0.69 | 0.82 | 0.12 |
| Tryptophan | 1.00 | 1.18 | 0.63 | 0.73 | 0.13 |
| Hydroxyproline | 1.00 | 0.97 | 0.94 | 0.81 | 0.04 |
| N-Acetylaspartate | 1.00 | 1.07 | 0.65 | 0.76 | 0.10 |
| 2-hydroxyglutaric acid | 1.00 | 1.15 | 3.48^*^ | 3.11^*€^ | 0.65 |
| Glycerol | 1.00 | 0.93 | 0.96 | 1.02 | 0.02 |
| Glycerol 3-phosphate | 1.00 | 2.13^*^ | 0.85 | 0.99^€^ | 0.30 |
| β-Glycerophosphate | 1.00 | 6.91^*^ | 0.26^*^ | 1.03^¥€^ | 1.55 |
| Glutarate | 1.00 | 1.65^*^ | 1.46 | 0.61^¥€^ | 0.23 |
| Adenine | 1.00 | 1.23 | 0.93 | 1.24 | 0.08 |
| Guanine | 1.00 | 1.77^*^ | 0.62 | 0.90^€^ | 0.25 |
| Uracil | 1.00 | 1.01 | 1.63^*^ | 1.42 | 0.16 |
| Thymine | 1.00 | 1.04 | 0.72 | 0.70 | 0.09 |
| Inosine | 1.00 | 1.16 | 1.01 | 1.13 | 0.04 |
| Adenosine | 1.00 | 0.51 | 0.80 | 1.21^€^ | 0.15 |
| Guanosine | 1.00 | 1.45 | 0.70 | 1.04 | 0.15 |
| Uridine | 1.00 | 1.16 | 0.90 | 1.41 | 0.11 |
| Pseudouridine | 1.00 | 0.64 | 0.58 | 0.79 | 0.09 |
| Cytidine | 1.00 | 1.12 | 0.46 | 0.50^€^ | 0.17 |
| Glutathione, reduced | 1.00 | 0.73 | 2.17^*^ | 1.26^¥^ | 0.31 |
| Glutathione, oxidized | 1.00 | 1.04 | 0.86 | 0.98 | 0.04 |
| UMP | 1.00 | 1.07 | 1.22 | 1.36 | 0.08 |
| UDP | 1.00 | 1.00 | 1.01 | 0.97 | 0.01 |
| UTP | 1.00 | 0.99 | 0.87 | 0.84 | 0.04 |
| UDP-glucose / UDP-galatose | 1.00 | 0.98 | 0.79 | 0.77 | 0.06 |
| UDP-glucuronic acid | 1.00 | 0.91 | 1.04 | 1.24 | 0.07 |
| UDP-N-acetylglucosamine/galctosamine | 1.00 | 0.87 | 1.23 | 0.91 | 0.08 |
| AMP | 1.00 | 0.79 | 1.08 | 0.88 | 0.06 |
| ADP | 1.00 | 1.01 | 0.99 | 0.88 | 0.03 |
| ATP | 1.00 | 1.00 | 1.02 | 0.94 | 0.02 |
| NAD^+^ | 1.00 | 1.06 | 1.36 | 1.08 | 0.08 |
| NADH | 1.00 | 0.67 | 0.89 | 0.76 | 0.07 |
| NADP^+^ | 1.00 | 0.81 | 0.89 | 0.58 | 0.09 |
| NADPH | 1.00 | 0.90 | 0.88 | 0.66 | 0.07 |
| CMP | 1.00 | 1.01 | 0.67 | 0.55 | 0.12 |
| CDP | 1.00 | 0.93 | 1.23 | 0.98 | 0.07 |
| CTP | 1.00 | 0.90 | 0.85 | 0.76 | 0.05 |
| CDP-Choline | 1.00 | 1.74^*^ | 1.06 | 0.74^€^ | 0.21 |
| CDP-Ethanolamine | 1.00 | 0.98 | 0.59 | 0.26^*^ | 0.18 |
| FAD | 1.00 | 1.12 | 1.35 | 1.04 | 0.08 |
| GMP | 1.00 | 1.48 | 0.91 | 1.09 | 0.12 |
| GDP | 1.00 | 1.33 | 0.71 | 0.78 | 0.14 |
| GTP | 1.00 | 0.94 | 0.83 | 0.71 | 0.06 |
| dGTP | 1.00 | 1.00 | 1.02 | 0.94 | 0.02 |
| GDP-Mannose | 1.00 | 1.21 | 0.89 | 0.83 | 0.08 |
| dTTP | 1.00 | 1.04 | 0.92 | 0.88 | 0.04 |
| IMP | 1.00 | 1.68^*^ | 3.03^*^ | 2.22^*¥^ | 0.43 |
| Nicotinamide Mononucleotide | 1.00 | 1.28 | 1.38 | 1.20 | 0.08 |
| Xanthine | 1.00 | 0.34^*^ | 2.03^*^ | 1.04^¥€^ | 0.35 |
| Choline | 1.00 | 4.66^*^ | 0.37^*^ | 1.07^¥€^ | 0.97 |
| Creatine | 1.00 | 1.08 | 0.93 | 0.95 | 0.03 |
| Phosphocreatine | 1.00 | 1.05 | 0.80 | 0.75 | 0.07 |
| Creatinine | 1.00 | 1.03 | 1.01 | 0.83 | 0.05 |
| N-Acetylglucosamine | 1.00 | 1.33 | 1.10 | 1.35 | 0.09 |
| Folate | 1.00 | 0.97 | 1.02 | 0.75 | 0.06 |
| Glucosamine-6-phosphate | 1.00 | 1.08 | 0.83 | 0.97 | 0.05 |
| GlcNAc-Phosphate | 1.00 | 1.23 | 1.18 | 1.89^*¥€^ | 0.20 |
| pRpp | 1.00 | 1.24 | 0.63 | 0.43^*€^ | 0.18 |
| Ornithine | 1.00 | 1.42 | 1.18 | 0.87 | 0.12 |
| Citrulline | 1.00 | 1.17 | 0.79 | 0.74 | 0.10 |
| Argininosuccinate | 1.00 | 0.55 | 7.11^*^ | 5.91^*¥€^ | 1.68 |
| N-Acetylglutamate | 1.00 | 0.91 | 0.74 | 0.59 | 0.09 |
| S-Adenosylhomocysteine | 1.00 | 0.74 | 0.83 | 0.75 | 0.06 |
| S-Adenosylmethionine | 1.00 | 1.15 | 0.79 | 0.81 | 0.09 |
| 2-methylcitrate | 1.00 | 4.92^*^ | 0.89 | 1.13^€^ | 0.98 |
| Carnitine | 1.00 | 1.10 | 1.24 | 1.20 | 0.05 |
| Methylguanine | 1.00 | 1.14 | 1.06 | 0.87 | 0.06 |
| 3-Indoleacrylic acid | 1.00 | 1.21 | 0.62 | 0.72 | 0.13 |
| Acetyl-Lysine | 1.00 | 1.09 | 0.54 | 0.66 | 0.13 |
| N,N-Dimethylarginine | 1.00 | 1.21 | 0.87 | 1.01 | 0.07 |
| Acetylcarnitine | 1.00 | 0.54 | 0.64 | 0.33^*^ | 0.14 |
| Methyladenosine | 1.00 | 0.77 | 1.21 | 0.74 | 0.11 |
| Cysteine-glutathione disulfide | 1.00 | 1.40 | 0.28^*^ | 0.44^*€^ | 0.26 |
| 2KIVA | 1.00 | 0.73 | 2.25^*^ | 1.44^¥€^ | 0.33 |
| Trigonelline | 1.00 | 0.83 | 1.28 | 0.93 | 0.10 |
| Methylmalonyl-CoA | 1.00 | 5.34^*^ | 0.10^*^ | 0.33^*€^ | 1.23 |
| 4-methyl-2-oxovalerate | 1.00 | 0.77 | 1.82* | 1.32 | 0.23 |
| Orotic acid | 1.00 | 1.13 | 2.99^*^ | 2.34^*¥€^ | 0.48 |
| Palmitate | 1.00 | 1.04 | 0.82 | 0.72 | 0.08 |
| Oleate | 1.00 | 0.96 | 0.90 | 0.99 | 0.02 |
| Stearate | 1.00 | 1.05 | 0.86 | 0.71 | 0.08 |
| Gamma-Aminobutyrate | 1.00 | 0.66 | 1.20 | 0.79 | 0.12 |
| Ophthalmate | 1.00 | 0.62 | 2.19^*^ | 0.98^¥^ | 0.34 |
| Erythritol | 1.00 | 1.01 | 1.21 | 0.89 | 0.07 |
| 5-Aminopentanoate/Methyl 4-aminobutyrate | 1.00 | 0.85 | 1.42 | 1.21 | 0.12 |
| Guanidinoacetate | 1.00 | 1.32 | 1.07 | 1.33 | 0.09 |
| N-formyl-L-methionine | 1.00 | 1.09 | 0.57 | 0.57 | 0.14 |
| Citicoline | 1.00 | 1.81^*^ | 1.00 | 0.83^€^ | 0.22 |
| Phosphoethanolamine | 1.00 | 0.58 | 0.24^*^ | 0.07^*^ | 0.21 |
| Phosphorylcholine | 1.00 | 1.11 | 0.93 | 0.88 | 0.05 |
| Cystathionine | 1.00 | 1.16 | 1.43 | 1.58^*^ | 0.13 |
| 1-Methyl-L-histidine | 1.00 | 1.41 | 0.66 | 0.87 | 0.16 |
| 3-Methyl-L-histidine | 1.00 | 1.41 | 1.07 | 1.01 | 0.10 |
| Carnosine | 1.00 | 0.96 | 1.06 | 0.88 | 0.04 |
| Aminoadipate | 1.00 | 0.96 | 0.68 | 0.42^*^ | 0.13 |
| Deoxycarnitine | 1.00 | 1.23 | 0.87 | 0.79 | 0.10 |
| Pipecolate | 1.00 | 1.00 | 0.83 | 0.52 | 0.11 |
| N,N,N-Trimethyllysine | 1.00 | 1.39 | 0.98 | 0.88 | 0.11 |
| Betaine | 1.00 | 1.17 | 0.77 | 0.79 | 0.09 |
| Ribitol/Xylitol | 1.00 | 1.18 | 1.49 | 1.48 | 0.12 |
| Hippurate | 1.00 | 0.98 | 1.03 | 0.77 | 0.06 |
| Sarcosine | 1.00 | 1.08 | 0.96 | 0.91 | 0.04 |
| Taurine | 1.00 | 1.20 | 1.18 | 1.14 | 0.05 |
| Hypotaurine | 1.00 | 1.53 | 0.90 | 1.18 | 0.14 |
| Riboflavin | 1.00 | 1.05 | 1.27 | 0.98 | 0.07 |
| 2-Hydroxy-4-(methylthio)butanoate | 1.00 | 0.99 | 0.65 | 0.69 | 0.09 |
| Allantoin | 1.00 | 0.83 | 1.14 | 0.99 | 0.06 |
| Citramalate | 1.00 | 1.11 | 0.63 | 0.73 | 0.11 |
| Pyridoxine | 1.00 | 0.94 | 1.05 | 0.82 | 0.05 |
| Benzoate | 1.00 | 1.03 | 0.89 | 0.85 | 0.04 |
| All data were analyzed with 2-way ANOVA followed by a Tukey post-hoc test. All data expressed as mean fold changes with calculated SEM for each metabolite using Row statistics procedure in Graphpad Prism. N=3; ^*^*p*<0.05 vs shScr. ^¥^*p*<0.05 vs shScr + αKG. ^€^*p*<0.05 vs shMPC2. | | | | | |
